# Development of an extended action fostemsavir lipid nanoparticle

**DOI:** 10.1038/s42003-024-06589-5

**Published:** 2024-07-30

**Authors:** Farhana Islam, Srijanee Das, Md Ashaduzzaman, Brady Sillman, Pravin Yeapuri, Mohammad Ullah Nayan, David Oupický, Howard E. Gendelman, Bhavesh D. Kevadiya

**Affiliations:** 1 Department of Pharmacology and Experimental Neuroscience, University of Nebraska Medical Center, Omaha, NE USA; 2 Department of Biochemistry and Molecular Biology, University of Nebraska Medical Center, Omaha, NE USA; 3 Department of Pathology and Microbiology, University of Nebraska Medical Center, Omaha, NE USA; 4 Department of Computer Science, University of Nebraska Omaha, Omaha, NE 68182 USA; 5 Center for Drug Delivery and Nanomedicine, Department of Pharmaceutical Sciences, College of Pharmacy, University of Nebraska Medical Center, Omaha, NE USA

**Keywords:** Translational research, Antivirals

## Abstract

An extended action fostemsavir (FTR) lipid nanoparticle (LNP) formulation prevents human immunodeficiency virus type one (HIV-1) infection. This FTR formulation establishes a drug depot in monocyte-derived macrophages that extend the drug’s plasma residence time. The LNP’s physicochemical properties improve FTR’s antiretroviral activities, which are linked to the drug’s ability to withstand fluid flow forces and levels of drug cellular internalization. Each is, in measure, dependent on PEGylated lipid composition and flow rate ratios affecting the size, polydispersity, shape, zeta potential, stability, biodistribution, and antiretroviral efficacy. The FTR LNP physicochemical properties enable the drug-particle’s extended actions.

## Abstract (web summary)

An extended action fostemsavir delivered to cells by lipid nanoparticles.

## Introduction

According to the Joint United Nations Program on HIV/AIDS (UNAIDS), up to 40 million people worldwide are infected with the human immunodeficiency virus (HIV)^1^. While viral suppression by antiretroviral therapy (ART) reduces disease-associated comorbidities and mortalities^2,3^, daily treatment requirements often negatively affect regimen adherence. Intermittent ART usage has led to drug resistance and accelerated disease^4,5^. Fostemsavir (FTR) was developed as a result. It is a prodrug developed for HIV infection in persons living with HIV (PLWH) who are infected with multi-drug resistant (MDR) viruses. Currently, FTR is used along with other antiretroviral drugs (ARVs) in infected people who are not successfully treated by other commonly used antiretroviral medications^6–10^. PLWH with medication intolerance and viral rebound are candidates for FTR therapy^11^. These include drug resistance, ARV toxicities, and drug-drug interactions^12,13^. FTR is an HIV attachment inhibitor that binds and inhibits HIV-1 gp120 attachment to the viral CD4 receptor^7,10,14–16^. The drug is a phosphonooxymethyl prodrug metabolized to temsavir (TMR) through alkaline phosphatase-mediated hydrolysis^7,17^. TMR prevents the earliest step in the HIV-1 life cycle^16,18^. After FTR binding to HIV-1 gp120, the conformational state is locked in the closed configuration as is seen in all CXCR4, CCR5, or dual tropic viruses^19,20^. This antiviral mechanism is novel^21,22^. TMR has adequate membrane permeability^23^ and no differences in TMR susceptibility following maraviroc or enfuvirtide exposures^24^. However, despite FTR’s unique pharmacological properties, its short half-life limits its efficacy. FTR use requires multiple dosing to achieve its therapeutic effects. The dosage regimen of the oral formulation is 600 mg twice daily^25^. This may burden patients who need to adhere to a strict dosing regimen. We posit that dosing reductions can improve compliance^26,27^. Improving the pharmacokinetic properties may also reduce inherent toxicities as FTR carries potential side effects, including heart rhythm abnormalities and hepatotoxicity^28^. Notably, improved drug delivery presents a means to extend the drug’s action. Lipid nanoparticles (LNPs) would enhance the formulation of FTR. Due to their nanoscale dimensions (generally 70 to 150 nm) and adjustable surface properties. LNPs can improve drug transport, making them appealing for clinical translation. LNPs form stable drug depots in CD4 + T and myeloid cells^29^, improving FTR’s residence time^30^. LNP biodistribution parameters can improve delivery facilitated by ligands for receptors on target cells such as tissue or cell target specific monoclonal antibodies, mannose or galactose.

Our data support that changing microfluidic input parameters and PEGylated lipid content can affect particle size, encapsulation, and biodistribution. Each can be adjusted during microfluidic assembly^31,32^. Multiple FTR formulations were developed by modifying the FRR and keeping the drug concentration constant. These affected the particle’s stability, shape, zeta potential, morphology, and polydispersity^33,34^. Thus, we find that the LNP itself affects the drug’s extended antiretroviral action.

## Methods

### Reagents

FTR was purchased from Amadis Chemical (Gongshu District, Hangzhou, Zhejiang, China). 1, 20 distearoyl-phosphatidylethanolaminemethyl-polyethyleneglycol conjugate 2000 (DSPE-PEG2000) was obtained from Corden Pharma International (Plank Stadt, Germany). 1,2-Dimyristoyl-rac-glycero-3-methylpolyoxyethylene (DMG-PEG 2000), 1,2-Dioleoyloxy-3-trimethylammonium propane chloride (DOTAP) and 1,2-Dioleoyl-sn-glycero-3-phosphoethanolamine (DOPE) were purchased from NOF corporation (Shibuya-ku, Tokyo, Japan). Cholesterol from Sigma-Aldrich (Saint Louis, MO, USA), Rhodamine B 1,2-Dihexadecanoyl-sn-Glycero-3-Phosphoethanolamine, Triethyl-ammonium Salt (rhodamine DHPE) from Invitrogen Molecular Probes (Oregon, USA). Ammonium formate, 99% from Acros Organics (USA), HPLC grade formic acid, chloroform, and HPLC grade methanol (Sigma-Aldrich, St. Louis, MO, USA), Phosphate buffered saline from Sigma-Aldrich. Dulbecco’s Modified Eagle Medium (DMEM) was purchased from Corning Life Sciences (Tewksbury, MA, USA), and Bovine serum albumin (BSA) was purchased from Thermo Fisher Scientific (Waltham, MA, USA). Fetal bovine serum (FBS) was purchased from Atlanta Biologicals (Flowery Branch, GA, USA) and 3-(4,5-dimethyl-2-thiazolyl)-2,5-diphenyl-2H-tetrazolium bromide (MTT) from Sigma-Aldrich. Alexa Fluor™ 680 Phalloidin (A22286) and DAPI (4’,6-Diamidino-2-Phenylindole, Dihydrochloride) were purchased from Thermo Fisher Scientific. The primary antibodies Rab7 (H-50) (sc-10767) and Lamp1 (H4A3) (sc-20011) used in the study were obtained from Santa Cruz Biotechnology, Inc. (Dallas, TX, USA) and Alexa Fluor™ 488 goat anti-rabbit (A11008) secondary antibody was purchased from Thermo Fisher Scientific. Monoclonal mouse anti-human HIV-1p24 (clone Kal-1) (sc-65918) and the polymer-based HRP-conjugated anti-mouse EnVision secondary (ref K4001) was purchased from Agilent Technologies (Santa Clara, CA, USA).

### LNP preparations

LNPs were prepared by either thin-film hydration^35,36^ or by microfluidic assembly^33,37,38^. FTR was incorporated into LNPs synthesized based on size and encapsulation efficiency. In the thin-film hydration method, DSPE-PEG2000, DOPE, cholesterol, DMG-PEG2000, and DOTAP with mole percent of 10.83; 11.25; 24.06; 2.78; 51.07, respectively, were taken in a round bottom flask and dissolved in chloroform. To evaporate the solvent and make a thin lipid film the lipid solution was taken under the vacuum in a rotary evaporator (Heidolph, Hei-VAP Precision, G3B Vertical, Schwabach, Germany). The solution was stirred at 200 RPM and the temperature was maintained at 40 °C to dry overnight in a vacuum desiccator. 100 mg of FTR were dissolved in 2 mL of milliQ water and the lipid film was hydrated with FTR solution by stirring for 30 min at 200RPM under bath sonication. To remove the free FTR, dialysis was performed overnight in 4000 mL of milliQ water using the Float-A-Lyzer® G2 Laboratory Dialysis Device with a molecular weight cutoff (MWCO) of 3.5–5 kDa (Spectra/Por®, Spectrum Laboratories, Inc., Rancho Dominguez, CA, USA). Finally, the FTR-LNP colloidal formulation was passed through a 0.45 µm hydrophilic syringe filter (Millex-HP Syringe Filter Unit, polyethersulfone, 13 mm, MilliporeSigma, Burlington, MA, USA) and further characterization such as size, polydispersity index (PDI), and zeta potential tested.

A microfluidic approach utilizing the NanoAssemblr® Ignite™ system (Precision NanoSystems Inc. Vancouver, Canada) was also employed. Lipids, with the same molar percentage ratios as in the thin-film method, were fully dissolved in 5 mL of ethanol. Simultaneously, 100 mg of FTR was completely dissolved in 5 mL of Milli-Q water. Subsequently, the 5 mL of FTR solution was equally distributed into 5 Eppendorf tubes, and their volumes were adjusted to 1.0, 1.5, 2.0, 3.0, and 4.0 mL by adding Milli-Q water. These varying solutions were intended for use in ratios of 1:1, 1.5:1, 2:1, 3:1, and 4:1 for FRR, respectively. Similarly, the 5 mL lipid solution was divided into 5 Eppendorf tubes. The lipid solution was loaded into a 1 mL syringe for each formulation, while the FTR solution was drawn into a 3 mL syringe for the LNP1, LNP2, LNP3, and LNP4 formulations. A 5.0 mL syringe was used for the LNP5 formulation. The formation of particles was achieved by passing the mixture of lipids and FTR solutions through a microfluidics chip (NanoAssemblr® Ignite™ NxGen Cartridge from Precision NanoSystems Inc). The flow rate was adjusted according to a particular ratio, which could be 1:1, 1.5:1, 2:1, 3:1, or 4:1, with a total flow rate of 12 mL/min. To remove the organic solvent and free drug components, overnight dialysis was performed using the Float-A-Lyzer® G2 Laboratory Dialysis Device with a molecular weight cutoff (MWCO) of 3.5–5 kDa (Spectra/Por®, Spectrum Laboratories, Inc., Rancho Dominguez, CA, USA) placed in beaker of 4000 mL of phosphate-buffered saline (PBS, pH 7.2; Sigma-Aldrich). Finally, the FTR-LNP colloidal formulation underwent filtration through a 0.45 µm hydrophilic syringe filter (Millex-HP Syringe Filter Unit, polyethersulfone, 13 mm, MilliporeSigma). The particle size, polydispersity index (PDI), and zeta potential were assessed via dynamic light scattering (DLS; Malvern Nano-ZS instrument, Worcestershire, UK). For Rhodamine labeled LNPs, we employed commercially available Rhodamine B-DHPE to label Rhodamine B within our LNPs. This labeling process was incorporated during the organic phase. Specifically, we included 1 mg of Rhodamine B-DHPE alongside other lipids in 1 mL of ethanol during the preparation of the organic phase. Following this step, LNP synthesis was consistent with the prior published reports with the exclusion of Rhodamine B^39–41^.

### Drug encapsulation

The encapsulation efficiency of FTR in the LNPs was measured by Ultraperformance Liquid Chromatography (UPLC) with an ultraviolet-visible detector system using calibration curves with known FTR standards. To prepare the standards, we dissolved 100 µg FTR in 1 mL methanol, then, with serial dilution, we made 10 standards 50 µg, 25, 12.5, 6.25, 3.125, 1.5625, 0.78125, 0.390625, and 0.1953 µg/mL.

To prepare the UPLC sample, 1 mL of 5% Triton-X was added to 5, 5, 7.5, 8.75, and 10 µL of LNP1, LNP2, LNP3, LNP4, and LNP5, respectively, in 5 different vials and stirred overnight. Then the samples were transferred to Eppendorf tubes and centrifuged at 14,000 x g for 30 min using centrifuge (5417 R, Eppendrorf, Germany). After that, the supernatant was collected, and the volume was adjusted up to 1 mL by adding HPLC-grade methanol. For each sample, two duplicates of two different dilutions were made. The chromatographic separation of 10 µL sample injections was accomplished using Kinetex® 5 µm C18 100 Å 150 ×4.6 mm LC Column (Phenomenex®, Torrance, CA, USA) using a 10-min gradient of mobile phase A (7.5 mM ammonium formate in Optima-grade water adjusted to pH 3.2 using formic acid) and mobile phase B (100% Optima-grade MeOH) at a flow rate of 1.0 mL/min. Initially, the mobile phase composition was 20% B for the first 5 min at which time it was increased to 45% B over 10 s and held constant for 4 min. Mobile phase B was then reset to 20% over 10 s and held for 3 min for equilibration. For this study, a Waters Alliance® e2695 Separation Module in conjunction with the Waters 2489 UV/Vis Detector (Waters Corporation- Milford, MA, USA) was used. The percentage of drug entrapped within the LNP formulation was calculated using the equation.

1 $${{{\rm{Encapsulation}}}}\; {{{\rm{efficiency}}}}\, ({{{\rm{EE}}}} \% )=\frac{{{{\rm{weight}}}}\; {{{\rm{of}}}}\; {{{\rm{the}}}}\; {{{\rm{drug}}}}\; {{{\rm{in}}}}\; {{{\rm{formulation}}}}* 100}{{{{\rm{initial}}}}\; {{{\rm{weight}}}}\; {{{\rm{of}}}}\; {{{\rm{drug}}}}\; {{{\rm{added}}}}}$$

### cryo-TEM imaging of LNPs

The cryo-electron microscopy (cryo-EM) evaluations were conducted at the University of Minnesota. Briefly, a 3 µL aliquot of FTR-LNPs was applied to glow-discharged R2/1 300 mesh Quantifoil TEM grids (Ted Pella, Inc.) and incubated for a half minute. The grid was subsequently blotted by a piece of filter paper and plunged into liquid ethane. The TEM grid was imaged using an FEI Tecnai 300 kV field emission gun (ThermoFisher Scientific) and a Gatan Summit K2 direct electron detector camera (Gatan, Inc. Pleasanton, CA USA)^42^.

### LNP size distribution by artificial intelligence (AI)

An AI-based supervised method was used to analyze the size distribution of LNPs from cryo-TEM images to obtain the semantic segmentation of the LNPs. The cryo-TEM images underwent annotation of all lipid nanoparticles using the image labeler tool in MATLAB. Subsequently, these annotated images were employed as labeled data to train the U-Net model for semantic segmentation of the lipid nanoparticles (LNPs). The U-Net architecture was employed for the LNP segmentation task, a commonly used semantic segmentation approach in medical imaging^43^. The resulting segmentation masks were then processed using the watershed algorithm to separate overlapping particles^44^. Next, topological structural analysis was performed on the output masks using the OpenCV Python libraries to identify the contours of each particle. The rectangular shape contours in the lower-left corner of the image were used to extract the scale bar, and the rectangle’s width in pixels was used as the relative scale measurement. Circled shape contours were identified for particle detection, and the enclosing circle boundary was calculated for each object. Since most particles were spherical, the enclosing circle was used to estimate the particle size. Enclosed circles within a specified radius range were selected for the desired particle objects, and their radii were measured to obtain the desired size distribution.

### AFM imaging

FTR-LNP samples were placed on a bare mica surface and incubated for 2 minutes. Following incubation, the samples were quickly rinsed with deionized water and then dried using a gentle stream of argon. Images were captured using the MultiMode Nanoscope IV system (Bruker Instruments, Santa Barbara, CA) operating in Tapping Mode under ambient conditions. Silicon probes RTESPA-300 (Bruker Nano Inc., CA, USA), with a resonance frequency of approximately 300 kHz and a spring constant of about 40 N/m, were utilized for imaging at a scanning rate of around 1 Hz. The images were analyzed using the FemtoScan software package (Advanced Technologies Center, Moscow, Russia), with particle size measurement conducted utilizing the enum feature.

### LNP-based drug release

In vitro drug release was completed using PBS (pH 7.4, representing physiological pH) and citrate (pH 4, representing late lyososomal pH) buffers. Slide-A-Lyzer™ MINI Dialysis Devices (3.5 kDa MWCO, ThermoFisher Scientific) were employed. Fourth five mL of each buffer was used in the tube, and 150 µL of FTR-LNPs were diluted with 850 µL of the respective buffer and then placed on top of the filter membrane tube of the dialysis device. They were put inside a rotary incubator at 200 RPM and 37 °C for 15 days.

At 1, 3, 6, 12, 24 h, and 2, 3-, 5-, 10-, and 15- day time points. 150 µL of sample was collected and replaced with appropriate fresh buffer. The collected samples were analyzed by UPLC using the EE% measurement method. From the initial amount of drug taken and the amount of drug obtained in the buffer at a specific time interval, cumulative percent of drug release was calculated where we considered the amount of sample (150 µL) collected and replaced with fresh buffer at each time point in the calculation. This experiment was replicated three times for each buffer.

3 $${{\rm{cumulative}}} \, \% \, {{\rm{drug}}}\; {{\rm{release}}}=\frac{({{\rm{amount}}}\; {{\rm{of}}}\; {{\rm{drug}}}\; {{\rm{obtained}}}\; {{\rm{in}}}\; {{\rm{the}}}\; {{\rm{buffer}}}+{{\rm{amount}}}\; {{\rm{removed}}}\; {{\rm{at}}}\; {{\rm{the}}}\; {{\rm{previous}}}\; {{\rm{time}}}\; {{\rm{point}}})\,* 100}{{{\rm{amount}}}\; {{\rm{of}}}\; {{\rm{drug}}}\; {{\rm{taken}}}\; {{\rm{initially}}}}$$

### Cell assays

#### Isolation and cultivation of human monocyte-derived macrophages (MDMs)

Monocytes were obtained through leukapheresis from donors who tested negative for HIV-1/2 and hepatitis B, with all human donors providing written informed consent for collecting and using their blood in this study. As part of this reporting summary, we provide information about the ethics approval institutional review board (IRB) approval and consent relevant to the “research conducted involving human participants. Any data relevant to biological material” was denied, precluding any known identifiers by our university’s “IRB oversight committee.” Subsequently, donor blood cells were purified by counter-current centrifugal elutriation. Monocyte-derived macrophages (MDMs) were cultivated in DMEM enriched with 10% heat-inactivated pooled human serum, 10 μg/mL ciprofloxacin, 50 μg/mL gentamicin, and 1000 U/mL equivalent of macrophage colony-stimulating factor for 7 days to stimulate the differentiation of monocytes into macrophages (MDMs)^45–47^. Once differentiated, the resulting MDMs were employed in FTR-LNP uptake studies, subcellular tracking experiments, and antiretroviral assays. The acquisition of human blood cells through leukapheresis from HIV-1/2 and hepatitis seronegative donors was approved by the UNMC Institutional Review Board. All ethical regulations relevant to human research participants were followed.

#### Cytotoxicity profiling

Cell viability assay for MDM was carried out using 3-(4,5-dimethylthiazol-2-yl)-2,5 diphenyltetrazolium bromide (MTT). MDM was used at a cell concentration of 8.0 × 10^4^ per well seeded in a 96-well plate. LNP1 and Rh-LNP1 concentrations from 0.078 μM to 100 μM were prepared and added to the cells in triplicate. After treatment for 24 h, MTT analysis was carried out at 490 nm wavelength using the method described previously^48^.

#### Cellular uptake by flow cytometry

Cellular uptake was carried out by flow cytometry. Based on the MTT result, 1.0 × 10^6^ MDM per well was treated with 200 µM Rh-LNP1 in 12-well plates for 4 and 10 h. After treatment, cells were washed with PBS three times. 500 µL PBS was added and cells were collected in flow tubes. Cells were centrifuged at 400 × g at room temperature. The supernatant was discarded, and cel/ls stained with LIVE/DEAD™ Fixable Blue Dead Cell Stain Kit (Thermo Fisher Scientific) and fixed with FACs fix (2% v/v formalin (Sigma-Aldrich) in PBS). The cells were analyzed by flow cytometry with appropriate controls. The fluorochrome combinations used for this experiment were Rhodamine B and LIVE/DEAD™ Fixable Blue stain. Compensation was performed using single-stained cells and unstained cells. Acquisition and analysis were performed on a BD LSRFortessa™ SORP Flow Cytometer, and BD FACSDiva 8.0 software, respectively (BD Biosciences, San Jose, CA, USA). Data were collected with a 355 laser (60.4 mW) and emission filter 427/25, and a 561 laser (150 mW) and emission filter 586/15. The gating tree was set as follows: FSC/SSC (represents the distribution of cells in the light scatter based on size and complexity, respectively) to FSC-A/FSC-H (excludes events which could be more than single cells) to LIVE gate (Fixable Blue negative, which represents viable cells) to FSC-A/Rhodamine B or Rhodamine B as a histogram. Biexponential scaling was used. Data was reported as % positive relative to unstained control and single stained LIVE/DEAD™ Fixable Blue stain. Mean and median fluorescence intensities were also reported.

#### Electron microscopy

For transmission electron microscopic tests, MDM were incubated in 12-well plates at 10^6^ cells/well for 8 h with LNP1 (100 μM based on FTR concentrations). Cells were washed two times with PBS after treatment and centrifuged at 650 × *g* for 5 min at 4 °C (Sorvall Legend RT centrifuge, Thermo Electron Corporation, Waltham, MA, USA). Cell pellets were immersed and fixed in a solution containing 2% glutaraldehyde and 2% paraformaldehyde (PFA) in 0.1 M Sorenson’s phosphate buffer (pH 7.2) for at least 24 hours at 4 °C. Following fixation, the samples were rinsed three times with phosphate buffer to eliminate any residual fixative. During processing, they were further post-fixed in a 1% osmium tetroxide aqueous solution for 30 minutes. Subsequently, the samples underwent dehydration using a gradient of ethanol concentrations (50, 70, 90, 95, 100%) and were treated with propylene oxide to serve as a transitional solvent between the ethanol and Embed 812 resin. The samples were then allowed to remain overnight in a 50:50 solution of propylene oxide and resin until all the propylene oxide had evaporated.

Afterwards, the samples were incubated in fresh resin for 2 hours at room temperature before final embedding. Polymerization then took place at 65 °C for 24 hours. Thin sections (90 nm), prepared using a Leica UC7 Ultracut ultramicrotome, were placed on 200 mesh copper grids, sequentially stained with 2% Uranyl Acetate and Reynolds Lead Citrate, and examined with a Tecnai G2 Spirit TWIN electron microscope (FEI, Houston, TX, USA) operating at an accelerating voltage of 80 kV. Digital images were acquired using an AMT digital imaging system^49,50^.

#### Confocal microscopy

Sub-cellular localization of Rhodamine stained FTR-LNP treated MDM was carried out by immunocytochemistry as previously described^51–54^. MDM were treated with Rh-LNP1 at 100 μM, and cells were washed with PBS and fixed with refrigerated 4% PFA. After, cells were washed three times with PBS. Cells were then treated with 0.25% Tween 20 for 15 min. Next, blocking solution (0.5 mL 100% Normal goat serum, 250 μL 10% Tween 20, 10% bovine serum albumin, 8.25 mL PBS) was added and kept on a rocker for 1 h. MDM were treated with primary antibodies against Rab7 for late endosomes and Lamp1 for lysosomes. Primary antibodies were diluted 1:250 v/v and incubated overnight with shaking at 4 °C as previously described^49^. MDM was then treated with secondary antibody Alexa Fluor™ 488 (Thermo Fisher Scientific) in an antibody solution (1:400) for 2 h at room temperature. F-actin was stained using Alexa Fluor™ 680 Phalloidin (Thermo Fisher Scientific). Nuclei were stained using DAPI (4’,6-Diamidino-2-Phenylindole, Dihydrochloride) with an emission at 461 nm (Thermo Fisher Scientific). Cells were imaged using a 63X oil objective on an LSM 800 confocal microscope (Carl Zeiss Microimaging, Inc., Dublin, CA, USA). Zeiss LSM 800 Image Browser AIM software version 4.2 was used to determine numbers of pixels and mean intensity of each channel.

#### Measurements of antiretroviral activities

ARV activity testing was done in HIV-1-challenged MDM. The study comprised of MDM treatments with LNP1, LNP5, empty LNP, and native FTR at 100 μM for 8 h. At 3, 6, 9, 12, and 24 h after treatment, the cells were challenged with HIV-1_ADA_ (a macrophage-tropic viral strain) at a multiplicity of infection (MOI) of 0.1 infectious particles/cell for 4 h according to previously established protocols^29,45,55^. Cells were fixed in 4% paraformaldehyde (PFA) at each timepoint. The fixed cells underwent blocking with 10% BSA in PBS, supplemented with 1% Triton X-100, at room temperature for 30 minutes. Subsequently, the cells were incubated with mouse monoclonal antibody targeting HIV-1p24 (1:100) for 3 h at room temperature. The binding of the HIV-1p24 antibody was visualized using the Dako EnVision+ System employing an HRP-labeled polymer anti-mouse secondary antibody and diaminobenzidine staining. Counterstaining of cell nuclei was achieved by treating the cells with 500 μL hematoxylin per well for 60 seconds. Bright field images were captured using a Nikon inverted bright field microscope with a 20× objective. For reverse transcriptase (RT) assay, media was collected 10 days post-infection. 10 μL of media from each well was plated in duplicate in 96 well plates. After incubation with appropriate solutions and harvesting of the media, TopCount® NXT^TM^ Microplate Scintillation and Luminescence Counter (Packard, Meriden, CT, USA) were used to read the results using TopCount NXT 2.5 software^56^.

### Animal studies

#### Mice

Male Balb/c mice (12 weeks old, 25 g body weight) were obtained from Charles River Laboratories (Stilwell, KS, USA) and accommodated in the UNMC Comparative Medicine animal facility adhering to the guidelines outlined in the Guide for the Care and Use of Laboratory Animals (National Research Council of the National Academies, 2011). We have complied with all named federal ethical regulations for animal use. This ensured the ethical treatment and utilization of laboratory animals in experimental research, as per protocol 22-029-08-EP. The housing followed the guidelines outlined by the Association for Assessment and Accreditation of Laboratory Animal Care (AAALAC). Approval for animal experimental protocols was obtained from the UNMC Institutional Animal Care and Use Committee (IACUC), ensuring alignment with the National Institutes of Health’s standards and ethical guidelines for treating laboratory animals in research. These animals were used to analyze FTR-LNP formulation biodistribution.

#### LNP biodistribution

Animals were administered a single intravenous dose of 100 µL or 200 µL of Rh-LNP1 and Rh-LNP5 formulations, respectively, using a 28 G × ½” needle via tail vein. Using the encapsulation efficiency percentages of Rh-LNP1 and Rh-LNP5 formulations, we determined the injection volume while maintaining the FTR dose consistent across both treatment groups. Following injection, animals were humanely euthanized, and organs were collected for fluorescence imaging.

The IVIS® Spectrum in vivo imaging system (PerkinElmer, Waltham, MA, USA) was employed to track the distribution of these two formulations at 30 min, 1, 2, 4, 6, 8, 10, and 12 h timepoints. IVIS can detect the fluorescence of Rhodamine (for rhodamine red, excitation = 571 nm and emission = 591 nm). Two groups of mice were injected intravenously (IV) with 100 µL Rh-LNP1 or 200 µL Rh-LNP5. The mice were sacrificed at the designated timepoints, and organs such as liver, spleen, lung, kidney, and heart were collected and imaged. The organs were imaged under IVIS using Living Image® 4.7.4 software and Living Image® 4.5 software was used for image analysis.

#### Statistics and reproducibility

For all studies, data were analyzed using GraphPad Prism 7.0 software (La Jolla, CA, USA) and presented as the mean ± standard error of the mean (SEM). In vitro experiments were performed with three biologically distinct replicates. Sample sizes were not based on power analyses. Animal studies included at least two animals at each time point per group, with the number of replicates listed.

### Reporting summary

Further information on research design is available in the Nature Portfolio Reporting Summary linked to this article.

## Results

The thin-film method was used for particle formulation in the first step of LNP development and testing. The process was chosen based on the ease of LNP formulation modification to target body organs and to improve the FTR’s encapsulated efficacy and pharmacokinetic properties. Ionizable lipids represent the principal constituent, effectively mediating drug delivery and the relationships between the size and shape parameters of the particles as evaluated by DLS and AFM tests. However, during these initial tests, its limitations became apparent. These limitations included non-uniform particle shape and size, lack of robustness, low encapsulation efficiency, the requirement for excess lipids, propensity for lipid aggregation, and heterogeneity within the nanoparticle formulation.

Based on the first phase experimental observations we adopted a microfluidic technology for our LNP delivery development. This method in comparison to the thin-film method provided more reproducibility with robust assay performance. The advantages of the microfluidic method included higher encapsulation efficiency, ease of rapid lipid mixing, scalability, and a reduced influence of mass transport. All the findings led to homogeneous LNP formulations and precise control over fluid dynamics. The latter included the ability to control the manufacturing parameters. In toto, the results showed LNPs with a precise and narrow size distribution, improved encapsulation efficiency, and limited lipid aggregation compared to the thin-film method. Five formulations were produced by microfluidics, and following physicochemical characterizations, the best candidate based on the physicochemical attributes was chosen for all the studies. In the animal and antiretroviral efficiency studies, we selected two formulations representing extremes in physicochemical characteristics: LNP1, which has a larger size and highest EE%, and another formulation, LNP5, exhibiting the smallest size and lower EE%. This approach evaluated whether these physicochemical attributes influence these formulations’ biodistribution and antiretroviral efficacy. The results of this process are developed below.

### Synthesis and physicochemical characterization of the FTR-LNPs

**Fig. 1:**
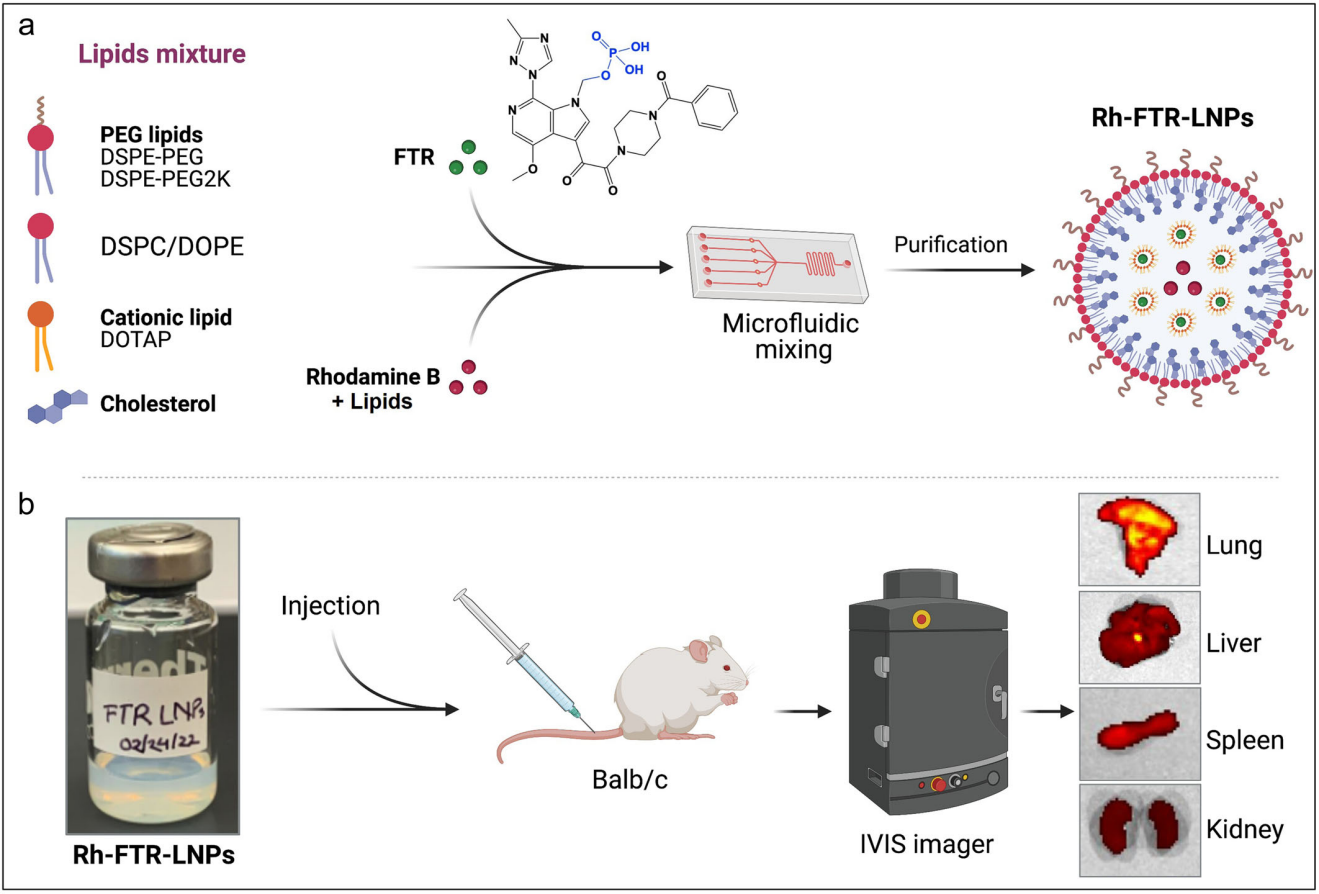
Preparation of FTR-LNPs. **a** Selected mixture of PEG lipids (DMG-PEG, DSPE-PEG), structural lipids (DOPE), cholesterol, and DOTAP was used as cationic lipid. First, the aqueous phase containing FTR fully dissolved in DI water and DHPE-Rhodamine B (RhB) was added in the lipid phase. For the preparation of Rh-FTR-LNPs, both phases were mixed with a microfluidic channel instrument (Ignite, Precision NanoSystems, Vancouver, Canada). After preparation, particles were purified by dialysis using a Float-A-Lyzer® G2 Laboratory Dialysis Device (MWCO: 3.5-5 kD, Spectra/Por®, Spectrum Laboratories, Inc., Rancho Dominguez, CA, USA) and sealed in a vial for animal injection. The bottom panel **b** shows a vial containing a colloidal homogenous suspension of Rh-FTR-LNPs. Prepared particles were injected in mice with Rh-FTR-LNPs, and at various time intervals in vivo biodistribution in organs such as liver, lung, and kidney were imaged using an IVIS imaging setup (PerkinElmer, Waltham, MA, USA).

The preparation of FTR-loaded LNPs is outlined in the pictorial study overview illustrated in Fig. 1. This includes the selection of lipid components that include PEG lipids, structural lipids, cholesterol, and cationic lipids. First, the aqueous phase contained FTR fully dissolved in water, with rhodamine B (RhB) in the lipid phase. Rh-FTR-LNPs were mixed with a microfluidic instrument. After preparation of particles, they were purified by dialysis, characterized, and sealed in vials for all subsequent animal injections.

The LNPs were initially made by thin-film hydration. The size of the LNPs was ∼130 nm and the PDI was ∼0.2. Both the size and PDI remained nearly unchanged over 30 days, showing good colloidal stability of the particles (Fig. S1a). We also synthesized empty LNPs without FTR and compared the size and PDI of the LNPs in the presence and absence of the drug. We found that the size and PDI of the empty LNPs were ∼120 nm and ∼0.23, respectively, which is not significantly different from the LNPs loaded with FTR and remained stable for 45 days (Fig. S1b). We have determined that thin film-manufactured LNPs exhibited a % encapsulation efficiency of 48.5. These values served as the secondary basis for additional investigations.

**Fig. 2:**
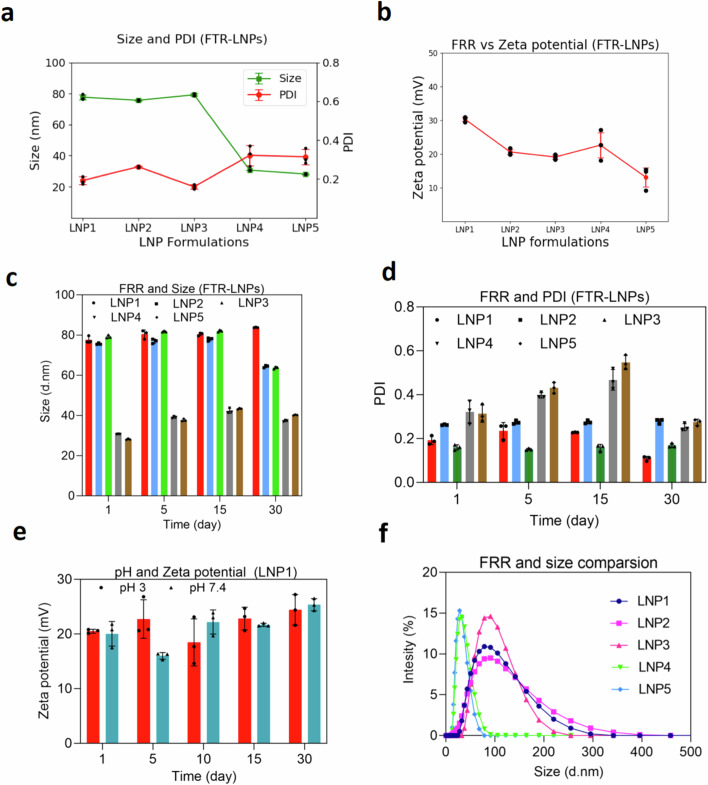
Process design parameters for FTR-LNP formulation optimization. **a** Size and PDI of different FTR-LNP formulations developed by microfluidics by changing FRR; LNP1, LNP2, LNP3, LNP4 and LNP5 have FRR of 1:1, 1.5:1, 2:2, 3:1 and 4:1, respectively. **b** Zeta potential of different FTR-LNP formulations at pH 7.0. **c**, **d** Stability of LNP formulations up to 30 days based on size and PDI measurements. **e** Zeta potential of LNP1 up to 30 days at two different pH, citrate buffer (pH 3.0) and phosphate-buffered saline (PBS; pH 7.4). **f** Comparison of size distributions of five FTR-LNP formulations prepared by variable FRRs. Data is presented as mean + SEM.

**Fig. 3:**
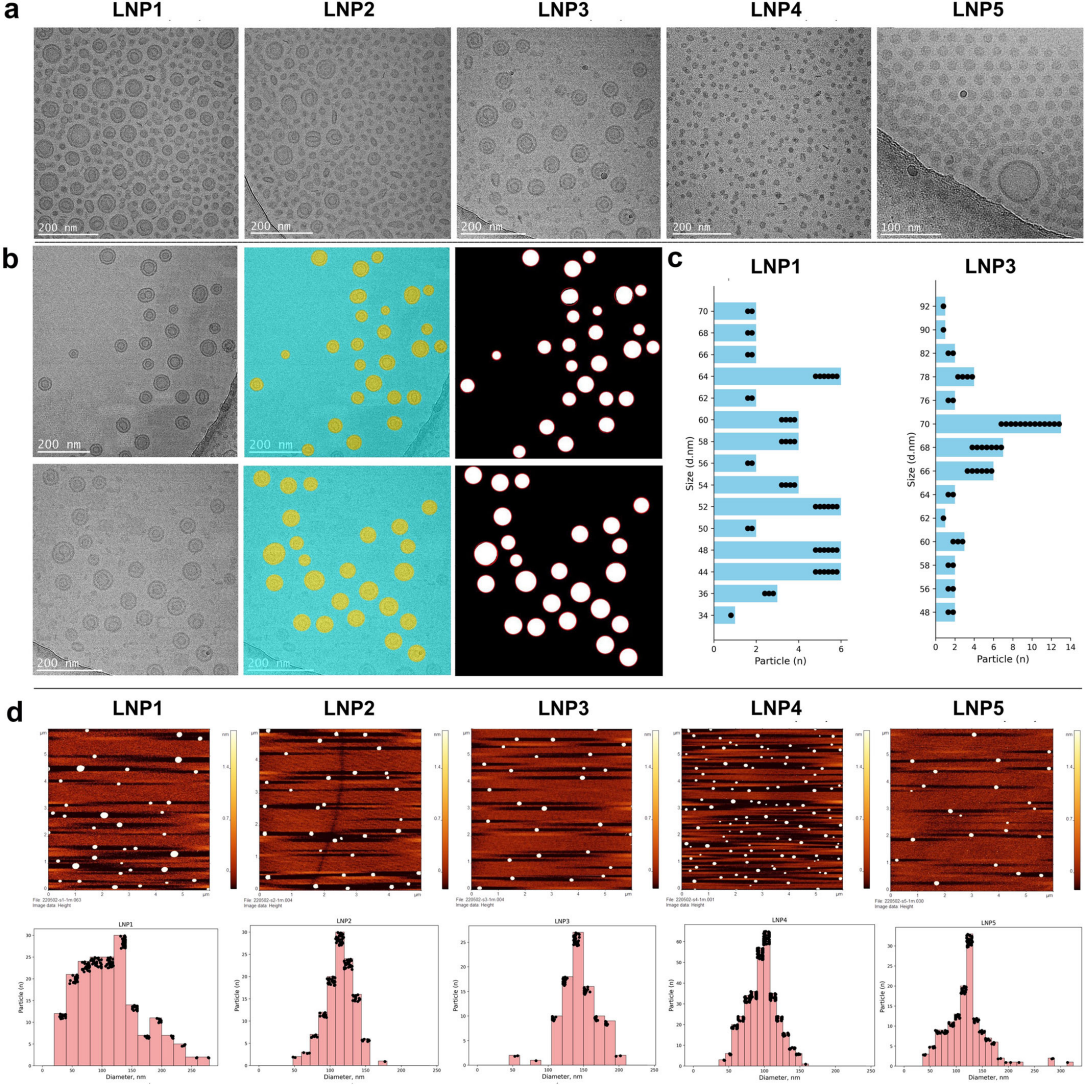
Microscopic images of FTR-LNPs with different flow rate ratios (FRR). **a** Cryo-TEM image of five different FTR-LNP formulations. **b** AI-based image processing is used to measure the size distribution of LNP1 (top) and LNP3 (bottom). The left column displays an original cryo-TEM image, the middle column shows the semantic segmentation output from the U-Net model predicting LNPs, and the right column illustrates the detected contours of the LNPs for size distribution. **c** Size distribution of LNP1 and LNP3 measured by image processing. **d** Topographic AFM images of FTR-LNPs (FTR-LNP distribution on mica substrate). Individual FTR-LNP size measurements with software and corresponding histograms of FTR-LNP size distribution in diameter (nm) and heights of nanoparticles to represent the morphological features of the particles.

We then synthesized particles by microfluidic assembly. This enabled us to produce five formulations by changing the flow rate ratio (FRR) (Table 1). All five formulations had the same lipid composition, and an FTR amount was used. In the five different formulations, the only variable was the volume of the aqueous phase. The five different FRRs used were 1:1, 1.5:1, 2:1, 3:1, and 4:1. We routinely employed dynamic light scattering (DLS) to acquire data regarding average particle size, size distribution, and polydispersity index (PDI) to investigate the impact of varying FRR. Changing this parameter influenced the size, PDI, zeta potential, and stability of the particles (Fig. 2a–d). At higher FRR (1:1, 1.5:1, and 2:1), we generated larger LNPs with an average size of 79.7, 76.1, and 80.1 nm, respectively, and with lower PDI measuring 0.19, 0.26, and 0.16, respectively. As the FRR decreases to 3:1, we see a decrease in the size of the LNPs with an average size of 30.6 nm and an increase in the PDI, measuring 0.32. Upon further dilution to an FFR of 4:1, the average size and PDI measured was 28.9 nm and 0.31, respectively (Fig. 2a). This suggests that after a certain level of dilution the average size and PDI of the LNPs becomes stable and it does not change with further increase of dilution of the aqueous phase. The zeta potential of these LNP formulations was in the range of +13.1 to +30.3 (Table 1 and Fig. 2b). Subsequently, the stability of these five LNP formulations was observed in relation to their size and PDI over a 30-day period. The average size of the formulations exhibited consistent stability throughout this duration (Fig. 2c). However, while the PDIs of LNP1, LNP2, and LNP3 remained steady, those of LNP4 and LNP5 exhibited fluctuations over the 30-day timeframe (Fig. 2d). The stability of the zeta potential of LNP1 measured at two different pH (pH 3 and pH 7.4) over 30 days was not significantly different (Fig. 2e). Observing the size distribution is important as it indicates the homogeneity of the formulation. The homogeneity of the particles is one of the determinant factors of the stability of the formulations. The size distribution of all five different formulations was plotted in a bell curve. A distinct single peak is observed in the size distribution analysis of all formulations (Fig. 2f). The corresponding low PDI values suggest monodispersity in these samples. Following this, we aimed to perform a morphological analysis of the LNPs based on cryo-TEM images. Subsequently, we compared the size distributions obtained through DLS with the data acquired through cryo-TEM. Figure 3a shows a representative cryo-TEM image of five different FTR-LNP formulations prepared using the microfluidic method. These images elucidate the diversity of sizes, shapes, and lamellar properties of the LNPs within a single formulation. Images of LNP1 and LNP2 reveal the presence of both bilayered and predominantly single-layered LNPs. While LNP3 exhibits a combination of bi- and single-layered LNPs, the proportion of single-layered LNPs is comparatively lower. In contrast, LNP4 and LNP5 have a single population of single-layered LNPs. Subsequently, LNP1 and LNP3 were chosen for size distribution analysis using AI (Fig. 3b left panel). Utilizing AI techniques, semantic segmentation masks were generated to detect LNPs in the cryo-TEM images of LNP1 and LNP3 (Fig. 3b, middle panel). In this representation, objects highlighted in yellow signify the detected LNPs, while the blue object represents the background. Subsequently, contours outlining the borders of the LNPs were identified for size distribution analysis through image processing techniques (Fig. 3b, right panel). LNP1 and LNP3 size distribution histograms derived from Fig. 3b are shown in Fig. 3c. Cryo-TEM images for FTR-LNPs and empty LNPs prepared by the thin-film hydration method were also used to observe the particle morphology and size distribution by AI and image processing techniques (Figs. S2a–e). Figure S2a shows the cryo-TEM images of FTR-LNPs and Fig. S2b shows cryo-TEM images of empty-LNPs. Both figures reveal a bilayered structure of the LNPs. Like the AI process discussed above, semantic segmentation masks were generated to detect LNPs in the cryo-TEM images of FTR-LNP (Fig. S2c). Subsequently, contours outlining the borders of the LNPs were identified for size distribution analysis through image processing techniques (Fig. S2d). Finally, the size distribution histogram is represented in Fig. S2e. Size distribution was again measured by atomic force microscopy (AFM) (Fig. 3d). It was found that FRR significantly affects particle size and distribution. Comparison of cryo-TEM and DLS data for LNPs with identical chemical compositions but differing molar ratios revealed notable disparities in average particle size, underscoring each technique’s distinct strengths and limitations. DLS analysis provided measurements of average sizes and PDI for a series of LNPs: LNP1 exhibited a size of 79.7 nm with a PDI of ~0.19, LNP2 had a size of 76.1 nm with a PDI of 0.26, LNP3 measured 80.1 nm with a PDI of 0.16, LNP4 showed 30.58 nm with a PDI of 0.32, and LNP5 displayed 28.50 nm with a PDI of 0.31 (Fig. 2a, f and Table 1). These DLS measurements relied on intensity-weighted hydrodynamic sizes, susceptible to influences from particle surface complexities, leading to a bias towards larger particles due to the relationship between scattering intensity and the square of the particle’s molecular weight. In contrast, cryo-TEM image analysis unveiled smaller average sizes, with LNP1 measuring 54.31 nm and LNP3 measuring 68.58 nm (Fig. 3c), considering *n* = 72 particles for LNP1 and *n* = 92 particles for LNP3 in per sample measurements. Consequently, the mean diameter obtained from cryo-TEM and DLS techniques aligns only under conditions of monomodal and monodisperse distributions. Examining the size distribution depicted in Figs. 2f and 3a, LNP1 exhibited a broad particle size, indicative of polydispersity, whereas LNP3 displayed a more uniform size. This variance in size distribution likely contributes to the greater disparity between DLS and cryo-TEM size measurements for LNP1 compared to LNP3.

**Table 1:** Flow rate ratio, size, PDI, and zeta potential analyses with 20 mg of FTR-LNPs

LNP formulations	Flow rate ratio (FRR)	Size (d.nm)	PDI	Zeta potential (mV)	EE%
LNP1	1.0:1	79.71	0.19	30.3	64
LNP2	1.5:1	76.09	0.26	20.3	26
LNP3	2.0:1	80.12	0.16	19.1	46
LNP4	3.0:1	30.58	0.32	22.6	49
LNP5	4.0:1	28.50	0.31	13.1	44

Moreover, consideration must be given to the number of particles per sample assessed in cryo-TEM evaluations. For AFM, the number of particles considered were *n* = 185 for LNP1, *n* = 121 for LNP2, *n* = 95 for LNP3, and *n* = 336 for LNP4 and *n* = 145 for LNP5, respectively (Fig. 3d histogram panel). Thus, we employed an orthogonal approach to particle size and PDI. Size distribution was also done for FTR-LNP formulations prepared by AFM’s traditional thin-film hydration (Fig. S3a–d). Here the number of particles analyzed was *n* = 173, where Fig. S3a and S3b show the morphology, and the size distribution of the FTR-LNP formulation is shown in a histogram in Fig. S3c, d.

### Drug release

**Fig. 4:**
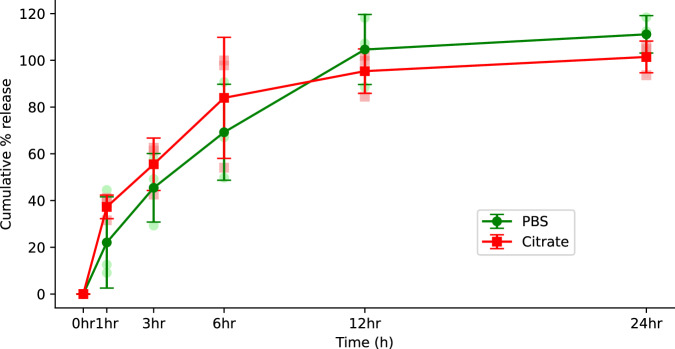
pH-dependent LNP1 drug release profiles. FTR was released into two distinct buffer solutions: phosphate-buffered saline (PBS) with a pH of 7.4 and citrate buffer with a pH of 4.0. FTR release tests were conducted under controlled conditions at 37 °C with continuous agitation at 200 rpm for up to 24 h. At specific intervals, precisely 150 μL of the test sample was withdrawn for subsequent analysis with UPLC at each designated time point. The experiment was done at *N* = 3 independent replicates for each buffer. Data is presented as mean + SEM.

The in vitro release of LNP1 is illustrated in Fig. 4. This was conducted with two buffers of varying pH (pH 4 and pH 7.4) over 15 days at 1, 3, 6, 12, and 24 h, timepoint (Fig. 4). These release profiles exhibit a notable burst effect during the initial phase (within 12 h), followed by a slower release phase observed at both pH^42,57^. In Fig. 4, it is evident that in PBS (pH 7.4) approximately 90% of the drug is released within the first 24 h, and with citrate buffer (pH 4) roughly 70% of the drug is released within the initial 24 h. Notably, within 5 days, almost 100% of the drug is released in both PBS and citrate buffer.

### FTR-LNP uptake and cytotoxicity

**Fig. 5:**
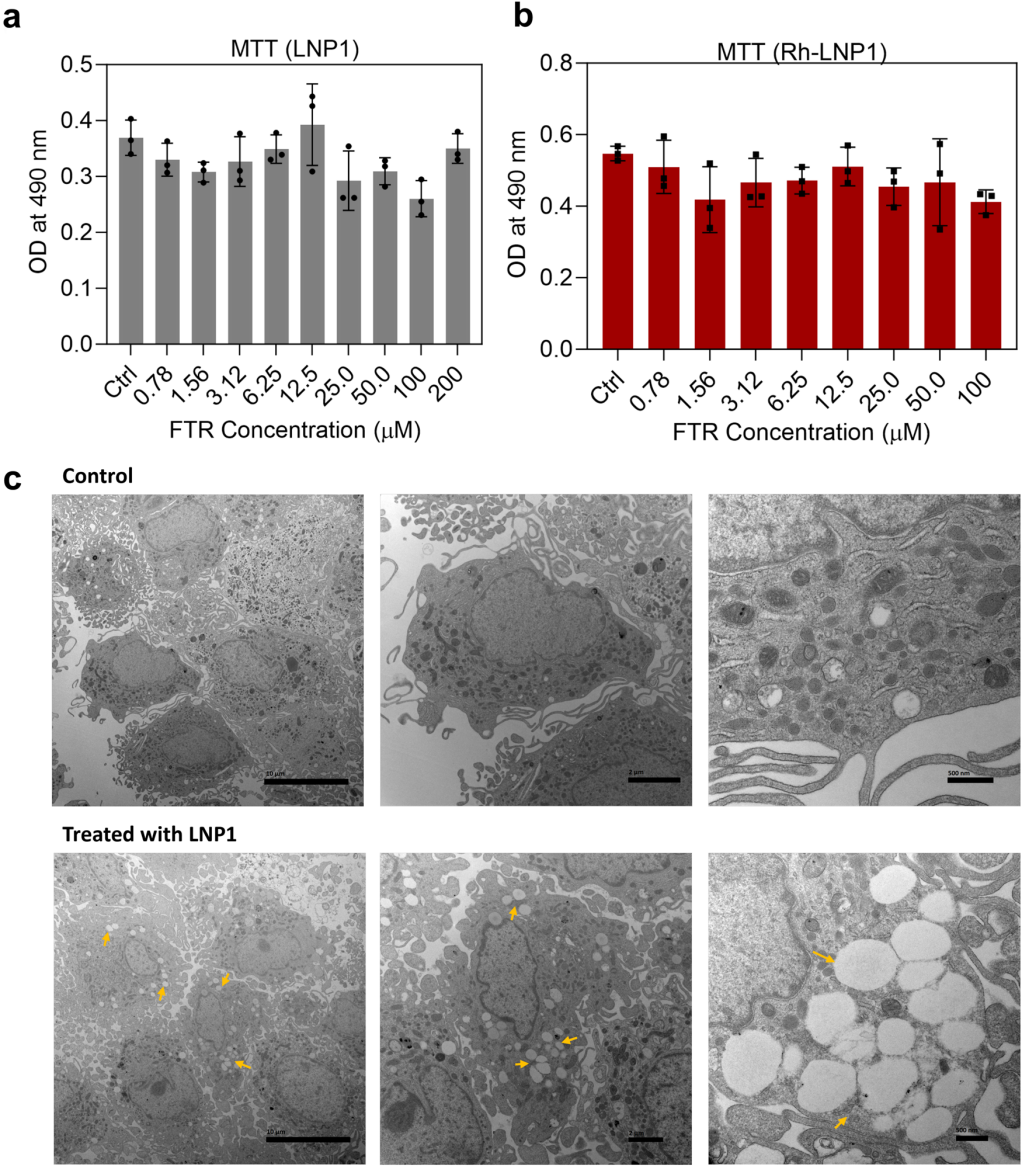
LNP toxicology and uptake profiles in MDM. **a** Viability test of FTR-LNP by MTT assay in macrophages (MDM) after 24 h incubation at 37 °C with untreated cells as negative control. MDM were treated with LNP1, varying the FTR concentration from 0.78 μM to 200 μM. **b** MDM were treated with Rh-FTR-LNP1, varying the FTR concentration from 0.78 μM to 100 μM. **c** Transmission electron microscopy (TEM) images of MDM treated with 100 μM LNP1 for four h at 37 °C incubation (bottom panel). The yellow arrows indicate LNP1 in the MDM. Untreated cells are considered as negative control (top panel). *N* = 3 biological replicates. From left to right, the scale bars measure 10 µm, 2 µm, and 500 nm. Data is presented as mean + SEM.

**Fig. 6:**
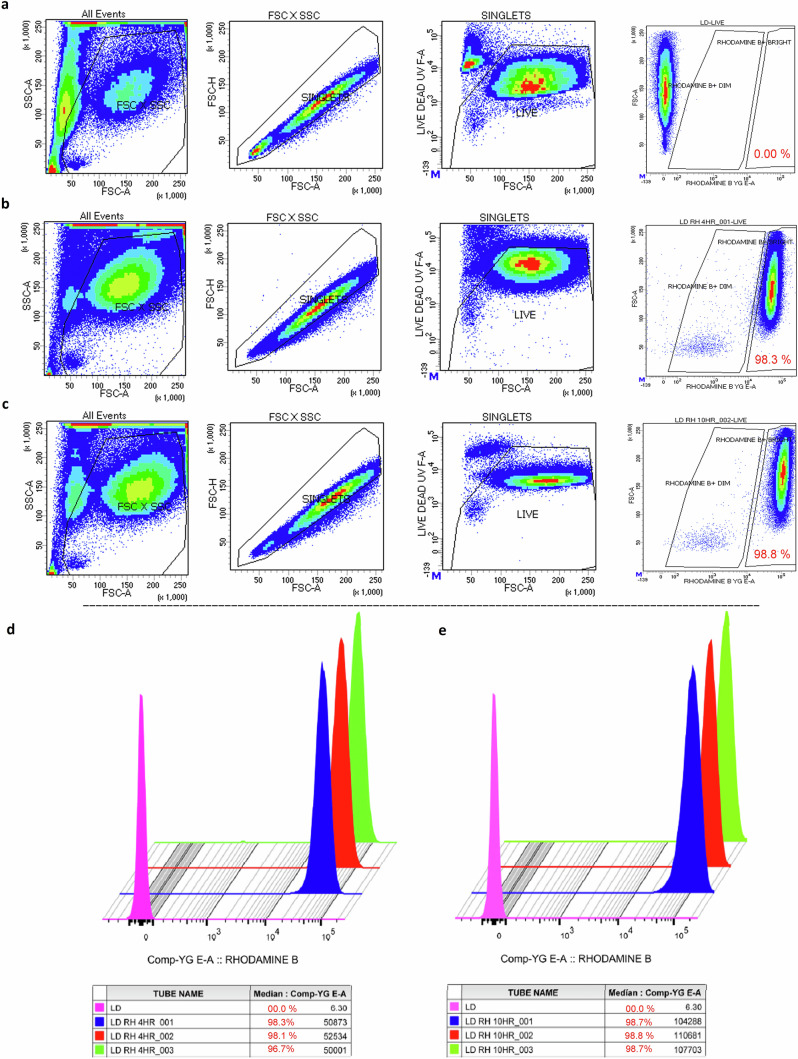
LNP1 cell uptake. Quantitative evaluation of LNP1 uptake by MDMs using flow cytometry. MDMs were treated with 100 μM LNP1 for 4 and 10 h at 37 °C. Cells were stained with live-dead fluorophore and then fixed with FACs fix. The percent of live MDMs was determined by live-dead staining of cells subjugated from single cells of the main forward-scatter and side-scatter density plots. The percent of LNP1 formulation and live double-positive cells (**b**, **c**) were gated based on live-stained untreated controls (**a**). **d**, **e** LNP1 uptake in MDM affirmed by the shift of population intensities for LNP1 at 4 and 10 h, respectively. Here, LD Live/Dead (pink), LD RH 4HR Live/Dead Rhodamine B 4 h (**d**), LD RH 10HR Live/Dead Rhodamine B 10 h (**e**); Blue, red, and green are three biological replicates at each time point.

Cellular toxicity and uptake studies of LNP1 were carried out in monocyte‐derived macrophages (MDM). MDM treated with LNP1 demonstrated no apparent cytotoxicity at concentrations up to 200 µM (Fig. 5a). For Rh-LNP1 up to 100 µM was not significantly cytotoxic (Fig. 5b) over 24 h using MTT vitality assay. The intracellular uptake of LNP1 was confirmed by transmission electron microscopy (TEM) images of MDM treated with 100 µM of LNP1 for 4 h. The results show a significant amount of LNP1 particles taken up in comparison to untreated MDM (Fig. 5c). A quantitative assessment of Rh-LNP1 uptake in MDM was done by flow cytometry in comparison to untreated (control) MDM. MDM were treated with 100 µM Rh-LNP1 formulation for 4 h and 10 h. The proportion of viable MDM was ascertained by isolating fluorophore-dim (live) cells within the sub-gate of single cells derived from the primary forward-scatter (FSC) and side-scatter (SSC) density plot. The percentage of LNP positive cells was determined by selecting the Rhodamine B bright cells sub-gated from the singlet live MDM. Up to 98.3% of gated singlet MDM demonstrated nanoparticle uptake at 4 h (Fig. 6b) and up to 98.8% uptake at 10 h (Fig. 6c). Figure 6a shows untreated (control) MDM. Rh-LNP1 uptake in MDM was affirmed by the shift of population intensities of Rh-LNP1 recorded cells in comparison to the untreated (control) cells (Fig. 6d, e). Cellular uptake of LNPs prepared by thin film hydration method in MDMs confirmed by confocal microscopy was showed in Fig. S4. This study showed that a significant amount of FTR-LNPs was taken up by the MDMs within 4 h.

### FTR-LNPs subcellular distribution

**Fig. 7:**
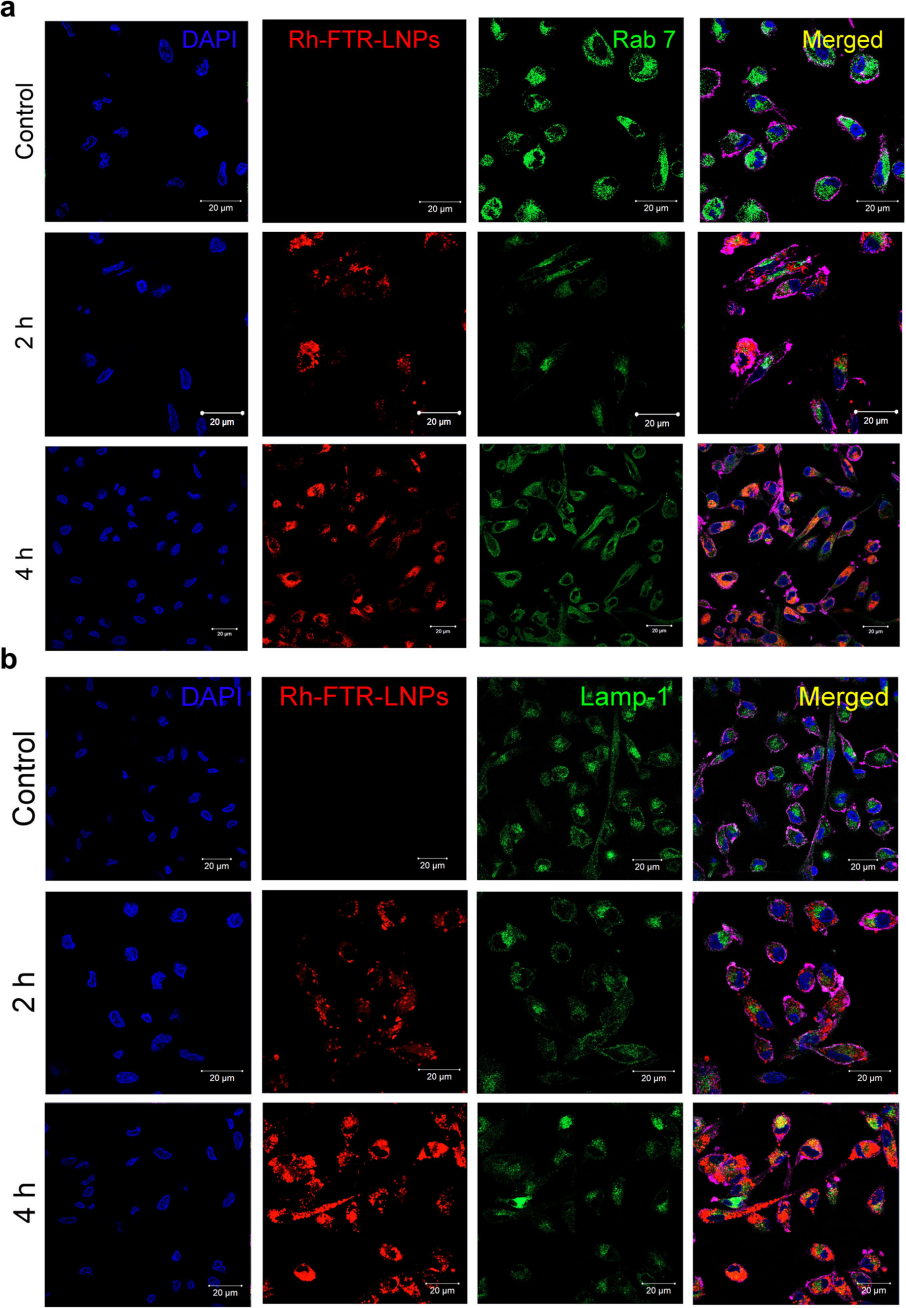
Trafficking of Rh-LNP1 in MDM subcellular compartments. Representative images of MDM treated with 100 μM Rh-LNP1 (red) after incubation for 2 and 4 h at 37 °C. Nuclei are stained with DAPI (blue). Cell membrane was visualized by staining for F-actin using Alexa Fluor™ 680 Phalloidin (magenta). **a** Treated cells were immunostained with primary antibodies Rab 7 (green) and captured with Alexa-Flour 488 as the secondary antibody. **b** Additional cell preparations were immunostained with primary antibodies Lamp-1 (green) and captured with Alexa-Flour 488 as the secondary antibody. Untreated cells were the negative control. MDM were imaged by confocal microscopy. Scale bar = 20 μm. *n* = 3 biological replicates.

Understanding the intracellular dynamics of LNPs is a significant concern for enhancing the efficiency of the payload delivery. Once LNPs encapsulating the payload are internalized by cells through endocytosis, they undergo a sequential process, first entering late endosomes and subsequently lysosomes, where they undergo degradation^58^. Hence, LNPs must possess mechanisms for escaping the endosomes and facilitating efficient payload delivery. Lipid composition of LNPs not only supports the creation of uniform nanoparticles and facilitate effective encapsulation of payloads, it also assists in cellular uptake, and encourages the escape of cargo from endosomes^59^. To monitor the fate of LNPs once taken up by MDM, we examined the subcellular co-localization of Rh-LNP1 using confocal microscopy. MDM were treated with 100 µM Rh-LNP1 for 2 h or 4 h and were immunostained for the late endosomal marker Rab7 (Fig. 7a) and lysosomal marker Lamp1 (Fig. 7b). The observed fluorescent intensity (red) directly corresponds to the intracellular concentration of LNPs. The results presented in both figures indicate that, during the initial 2 h incubation with Rh-LNP1, cellular uptake of LNPs occurs, with relatively increased concentrations observed after 4 h incubation. It was revealed that Rh-LNP1 some extend exhibit localization in late endosomes 4 h timepoints (Fig. 7a). At the 2 h timepoint, Rh-LNP1 are absent in lysosomes. Subsequently, at the 4-h timepoint, only a marginal quantity of LNPs were found within the lysosomes (Fig. 7b). A substantial amount of LNPs were observed intracellularly but not localized within the lysosomal compartments, suggesting that Rh-LNP1 can avoid degradation in the lysosomes.

### FTR-LNP biodistribution in mice

**Fig. 8:**
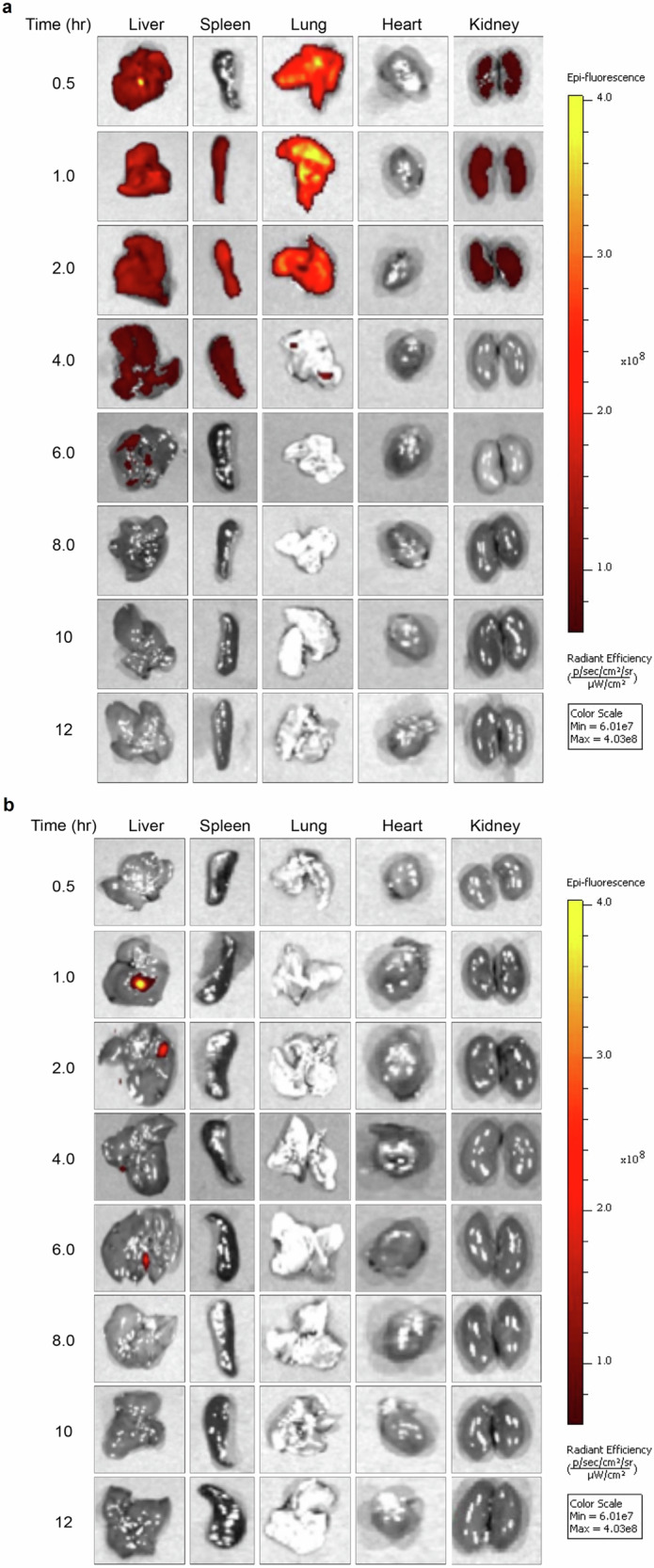
Biodistribution of FTR-LNP formulations. Experiments were performed in male Balb/c mice by in vivo imaging system (IVIS). 100 and 200 μL Rh-LNP1 and Rh-LNP5 formulations, respectively, were administered. Intravenous injections were used to assay the biodistribution of both LNP1 and LNP5. At 30 min and 1, 2, 4, 6, 8, 10, and 12 h, mouse liver, spleen, lung, heart, and kidney organs were collected. Organs were imaged under IVIS, and the fluorescence signal was recorded in FTR-LNP at excitation and emission of 571 and 591 nm, respectively. **a** Mice administered Rh-LNP1 and fluorescence signal in different organs. **b** Mice administered Rh-LNP5 and fluorescence in different organs. Images were analyzed using Living Image® software. *N* = 2 for each time point and treatment group.

We next evaluated the biodistribution of the LNP formulations in animals. To track the distribution, male Balb/c mice were administered intravenously with Rh-LNP formulations and ex vivo fluorescence imaging was done using PerkinElmer’s IVIS® optical imaging system, where fluorescently labeled exosomes or liposomes can be examined in tissues^60^. In our study, two groups of male Balb/c mice were administered with 100 µL Rh-LNP1 or 200 µL Rh-LNP5. Mice were euthanized 30 min, 1, 2, 4, 6, 8, 10, and 12 h after injection and organs were excised to capture the distribution of FTR containing LNP. Figure 8a reveals that for Rh-LNP1 treated mice, fluorescence was first detected at early timepoints, with the earliest signal observed at 30 min. At the 30-min timepoint, strong fluorescence was noted in the lung, followed by the liver, and some fluorescence was also observed in the kidney. By 1 h, along with lung, liver and kidney, fluorescence had become apparent in the spleen as well. Notably, the 2 h timepoint revealed relatively strong fluorescence in the spleen compared to any other timepoint. At the 4 h timepoint, minimal fluorescence was observed in the liver and spleen and by the 6 h timepoint, no fluorescence indicative of Rh-LNP1 was detected in any organ, suggesting the circulation of Rh-LNP1 out of the body by that time. These findings suggest that the Rh-LNP1 formulation exhibited distribution across multiple organs, with the primary accumulation occurring in the lung, liver, and spleen during the early timepoints. However, for the Rh-LNP5 formulation, there was no observed distribution at any timepoint (Fig. 8b).

### Antiretroviral activities

**Fig. 9:**
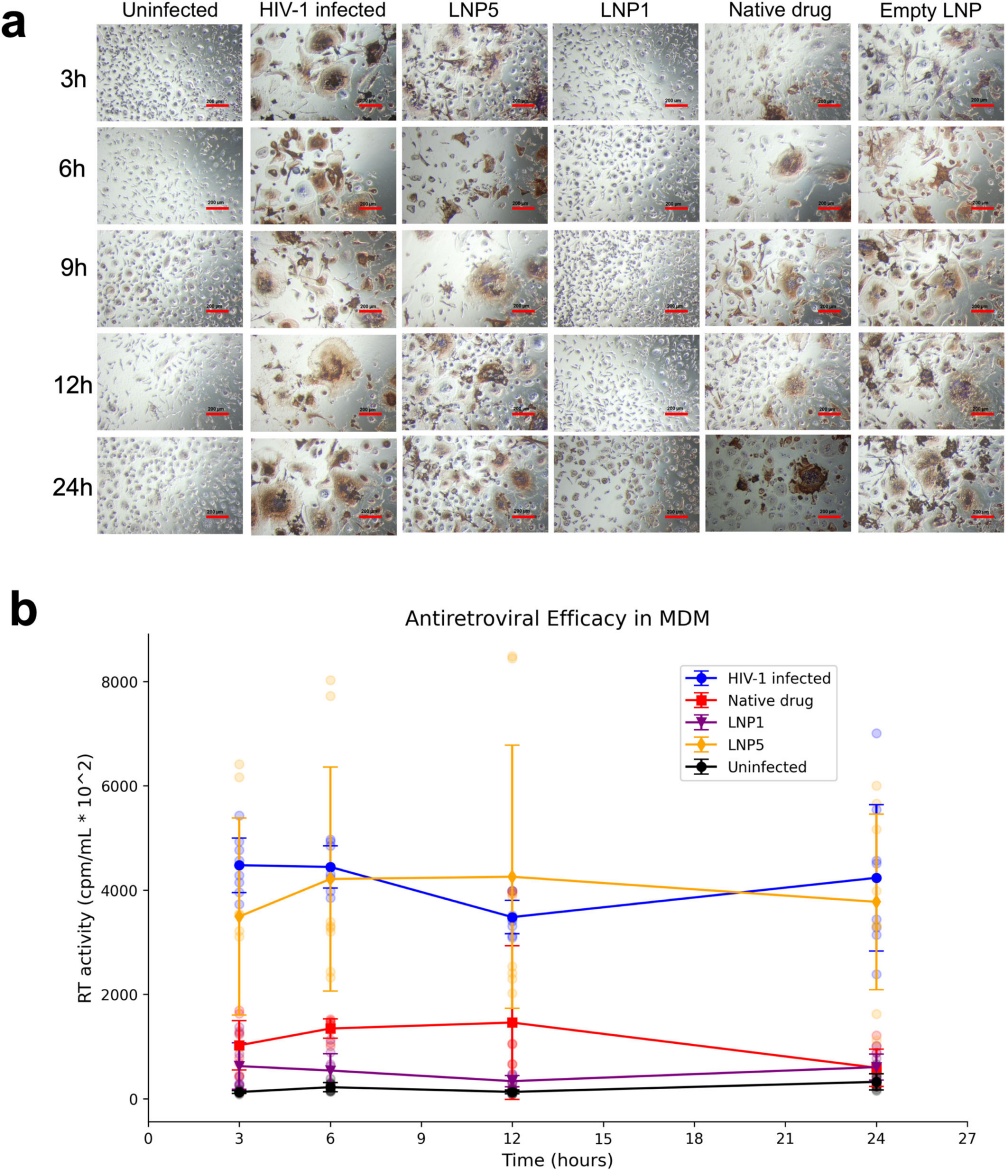
Antiretroviral activities of LNP1 and LNP5 formulations. **a** Antiretroviral responses were recorded by HIV-1p24 immunostaining after HIV-1_ADA_ challenge at a multiplicity of infection (MOI) of 0.1 infectious virions/cell in MDM cultured for 7 days. Following an 8 h administration of drug given at 100 μM LNP1, LNP5, or free fostemsavir to MDM, viral antigen staining was assessed—empty LNP containing no antiretroviral drug served as a control. Representative images were taken at 20X magnification. **b** Reverse transcriptase (RT) activities were determined in culture fluids from 3 to 24 h. *N* = 4 biological replicates. Data is presented as mean + SEM.

To evaluate and compare the antiretroviral activity of LNP formulations, we assessed HIV-1 RT activity and HIV-1p24 antigen expression in MDM. This assessment followed a single administration of 100 µM formulation to MDM, which was then followed by a series of HIV-1_ADA_ challenges at 3 h intervals over a 24 h period. We compared the efficacy of LNP1 and LNP5 formulations and used native FTR as a control. As depicted in both Fig. 9a (HIV-1p24 antigen expression) and Fig. 9b (HIV-1 RT activity), it is evident that both native FTR and LNP5 demonstrated viral replication at a 100 µM FTR dose starting from the 3 h to 24 h. In contrast, the LNP1 formulation protected MDM against HIV-1 challenge, beginning as early as 3 h and continuing up to 24 h after treatment with the same 100 µM dose.

## Discussion

The development of LNPs as a drug delivery platform is notable. LNPs are part of a family of solid lipid nanoparticles, nanostructured lipid carriers, and nanoemulsions, which have received consideration for antiretroviral drug (ARV) delivery^61^. LNPs improve ARV stability and facilitate biodistribution, including the lymphatic and central nervous systems. The particle size and LNP manufacturing improve efficiency, stability, and reduce immunogenicity. Such advantages revolve around the robustness of the formulation^32,62,63^. Thus, LNP ARV delivery presents both opportunities and challenges.

Loading hydrophilic FTR into LNPs is the first step to improving current FTR administration regimens. Formulation modifications may also improve drug toxicity. For example, the most frequently reported FTR adverse effect is nausea. FTR side effects can be even more notable as patients with hepatitis B or C coinfection commonly develop elevations in liver enzymes^12,64^. The ability of LNPs to transport drugs is highlighted by their ability to reduce the amount of administered medicines to achieve their desired therapeutic effect^65^. Notably, encapsulating FTR within an LNP can deliver the drug directly to the target cells or tissues, minimizing the amount of circulating drug^66^. LNPs can also improve the solubility and stability of drugs, which can further reduce toxicity^65,67^. LNPs help to solubilize drugs and protect them from degradation. In addition, LNPs can be designed to target specific cells, which can further reduce toxicity by minimizing the exposure of non-target tissues to the drug. We used the thin-film method to formulate LNPs to test the proof-of-concept experiments. We subsequently analyzed the LNPs using DLS and AFM to determine particle size and shape. Because the method produced particles with non-uniform shape, size, and robustness, microfluidic technology was developed as a replacement. Microfluidics demonstrated superior performance in the pharmacokinetic LNP profiles and was substituted in subsequent testing and analyses completed in this study.

The size measurement of the same LNPs is commonly higher when measured using DLS than cryo-TEM due to the different principles and conditions of these techniques. Consequently, particles may appear larger in DLS measurements. On the other hand, cryo-TEM or AFM provide direct imaging of particles in their dry or frozen state, respectively, and generally yield smaller size measurements^68–70^. Such a discrepancy arises because DLS captures the hydrodynamic diameter, encompassing the particle and the solvation layer. A study demonstrated that cryo-TEM, DLS, and zeta potential measurements can be used to characterize LNPs encapsulated small interfering RNA (siRNA). In the prior work, DLS measurements resulted in larger particle size values than cryo-TEM^41^. In contrast, AFM offers a topographical depiction of the nanoparticle, providing precise measurements of its physical dimensions on a surface when the sample is dry. However, cryo-EM offers advantages in visualizing the particle’s morphology, while DLS provides bulk assessments. AFM showed larger particle sizes across all formulations than cryo-TEM and DLS. LNP particle size affects cellular internalization, biodistribution, and LNP clearance^71^. FRR and total flow rate (TFR) influence encapsulation efficiency. Prior works showed that optimizing these parameters is essential for tailoring LNPs for drug delivery^72,73^. LNP1, LNP2, and LNP3 exhibit relatively similar sizes than LNP4 and LNP5. However, they differ significantly in their PDIs. LNP1 demonstrates the lowest PDI (~0.19), indicating a more narrow and uniform size distribution than LNP2 and LNP3. LNP1 has a more homogeneous particle size, which supports consistent therapeutic efficacy and robust tissue biodistribution. In contrast, LNP4 and LNP5, despite being smaller, have higher PDIs, indicating a broader size distribution and limited tissue distribution, potentially affecting long-term stability. They may be prone to aggregation or degradation followed by the release of drug substance from drug product^74^. LNP1 to LNP3, lower PDIs indicated a more uniform particle size distribution. This proved crucial for consistent LNP performance. Understanding the implications of PDI on the size distribution of LNPs is vital for optimizing their design^75^.

The FTR release profile of a drug product is a critical factor in determining its efficacy and safety^76^. In the case of our study LNP1, the fact that almost 90% of the drug is released within the initial 24 hours, this characteristic can still have potential advantages and implications. This rapid release of the FTR can lead to a quick onset of action. The successful delivery of ART therapeutics is highly dependent on the ability of LNP-delivered drugs to escape from endosomes and reach the cytosol. Despite the potential of LNPs as delivery vehicles, research indicates that only a minor fraction of the drug cargo successfully escapes the endosomes. Most are trapped within these cellular compartments upon endocytosis^77^. Understanding the factors that influence LNP endosomal escape is vital for enhancing the efficacy of LNP-based therapeutic delivery systems^78^. Our study observed that cells treated with Rh-LNP1 exhibited partial co-localization with endosomal markers, mainly when markers for late endosomes (Rab 7) and lysosomes (LAMP-1) were employed. This suggested that Rh-LNP1 is sequestered, highlighting the need for significant optimization of our lipid composition to facilitate a more efficient endosomal escape pathway, thereby improving therapeutic outcomes. The fate of endocytosed LNPs and their subsequent escape from endosomes is determined by a complex interplay of factors. These include the lipid composition and nanostructure of the LNPs, the nature of the encapsulated drug, and the physicochemical environment within the endosomes. Notably, the acidic pH within endosomes can influence the fusion of LNPs with the endosomal membrane, which is a prerequisite for drug release into the cytosol^78^. Our findings highlight the importance of meticulously designing LNP systems to overcome some of the extended barriers to endosomal entrapment. By tailoring the lipid composition and nanostructure and considering the specific interactions between LNPs and the endosomal membrane, we can develop more effective strategies for delivering ART therapeutics and potentially other drugs that require intracellular targeting.

LNPs have been associated with changes in liver enzymes and body weight, indicating a possible induction of hepatotoxicity. Studies have also revealed increased interferon (IFN) type I responses and related pathways systemically, with connections to Th1 cytokines like interleukin 2, IFNs, and tumor necrosis factor. However, the specific cargo carried by LNPs, such as drug substances, can mitigate these effects. For instance, different LNPs may impact innate immune responses differently, as observed with certain antiretroviral drugs, potentially counterbalancing any inherent toxicities of the particles themselves. Despite these considerations, LNPs are generally safe for liver health^79^.

In summary, microfluidics yielded LNPs with precise and narrow size distribution, enhanced encapsulation efficiency, and minimal lipid aggregation compared to thin-film methods. We developed five formulations, selecting LNP1, with the largest size and highest encapsulation efficiency, and LNP5, with the smallest size and lower encapsulation efficiency, for further studies. The LNP1 formulation was detailed for its physicochemical properties, biodistribution, and antiretroviral efficacy. These experiments affirmed the LNP performance.

In the future, using a spectrum of lipids or lipid-like molecules to create carriers is even more efficient at drug delivery. This could involve designing carriers with specific surface properties that facilitate specific cell uptake. Optimizing drug encapsulation or by incorporating newer techniques for drug loading drugs. Targeted delivery can also be achieved by decorating the surface of LNPs with specific ligands that bind to receptors on the target cells. Combining multiple drugs within LNPs could improve therapeutic outcomes. With its complete drug release within 24 hours, the developed FTR LNP formulation presents a promising opportunity to enhance patient compliance, particularly for individuals who may have difficulty adhering to a twice-daily oral tablet regimen^5^. In conclusion, research and development in the lipid-based drug delivery space could significantly improve efficacy and safety.

### Supplementary information

Supplementary Information

Description of Additional Supplementary Materials

Supplementary Data

Reporting Summary

## Data Availability

We included a zip file titled “Supplementary data.zip,” which contains the Excel file with individual data points added. The data that support the findings of this study are available from the corresponding authors upon reasonable request.

## Code availability

The custom computer code used in the “LNP Size Distribution by Artificial Intelligence (AI)” section of this manuscript is publicly available. The code can be accessed at the following GitHub repository: https://github.com/asadayon/LNP-size-Distribution. The repository is archived with a DOI provided by Zenodo: 10.5281/zenodo.11540200, ensuring permanent availability.
