# Supplementary material for: Development of an extended action fostemsavir lipid nanoparticle
Source: Commun Biol. 2024 Jul 30;7:917. doi: 10.1038/s42003-024-06589-5 (PMC11289258; doi:10.1038/s42003-024-06589-5)
Supplement: Supplementary file 1 — Supplementary Information [file 42003_2024_6589_MOESM1_ESM.pdf]

Supplementary Information

Development of an extended action fostemsavir lipid nanoparticle

Farhana Islam[1,2], Srijanee Das[1,3], Md Ashaduzzaman[4], Brady Sillman[1], Pravin Yapuri, Mohammad Ullah Nayan[1], David Oupický[5], Howard E. Gendelman1,3*, and Bhavesh Kevadiya[1]

[1]*Department of Pharmacology and Experimental Neuroscience, University of Nebraska Medical Center, Omaha, NE, USA*

[2]*Department of Biochemistry, University of Nebraska Medical Center, Omaha, NE, USA*

[3]*Department of Pathology and Microbiology, University of Nebraska Medical Center, Omaha, NE, USA*

[4]*Department of Computer Science, University of Nebraska Omaha, Omaha, NE, 68182, USA*

[5]*Center for Drug Delivery and Nanomedicine, Department of Pharmaceutical Sciences, College of Pharmacy, University of Nebraska Medical Center, Omaha, NE, USA*

Corresponding Author: Howard E Gendelman (for review, scientific content, and publication) email: hegendel@unmc.edu; phone 402 559 4044

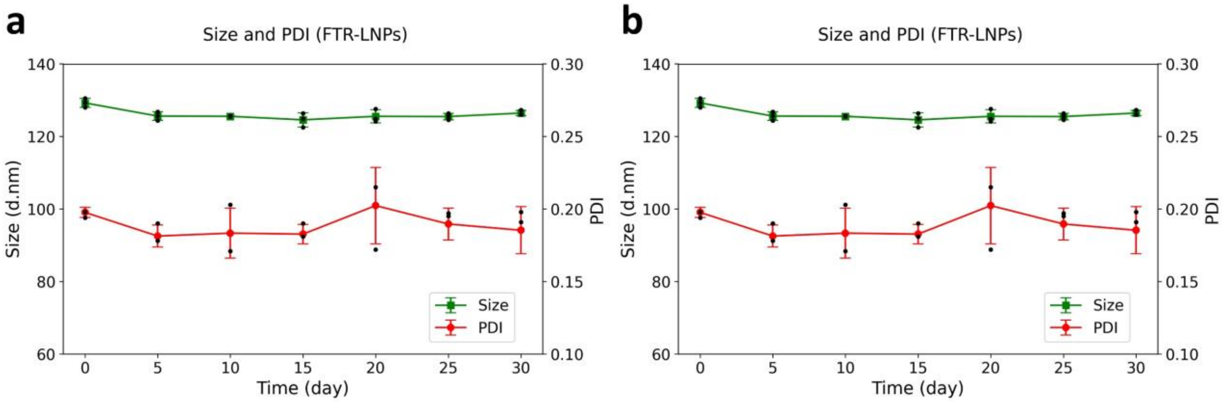

Supplementary Figure 1. Thin-film hydration LNP synthesis measurements. LNPs were prepared using the conventional thin film hydration technique, which involved the formation of a lipid thin film using a rotary evaporator. Subsequently, this lipid film was subjected to hydration using either FTR dissolved in milliQ water **a** or milliQ water alone **b**. The stability of FTR-LNPs was assessed by measuring their size and PDI over 30 days. The stability of empty-LNPs, devoid of FTR, was similarly monitored by measuring their size and PDI over 30 days.

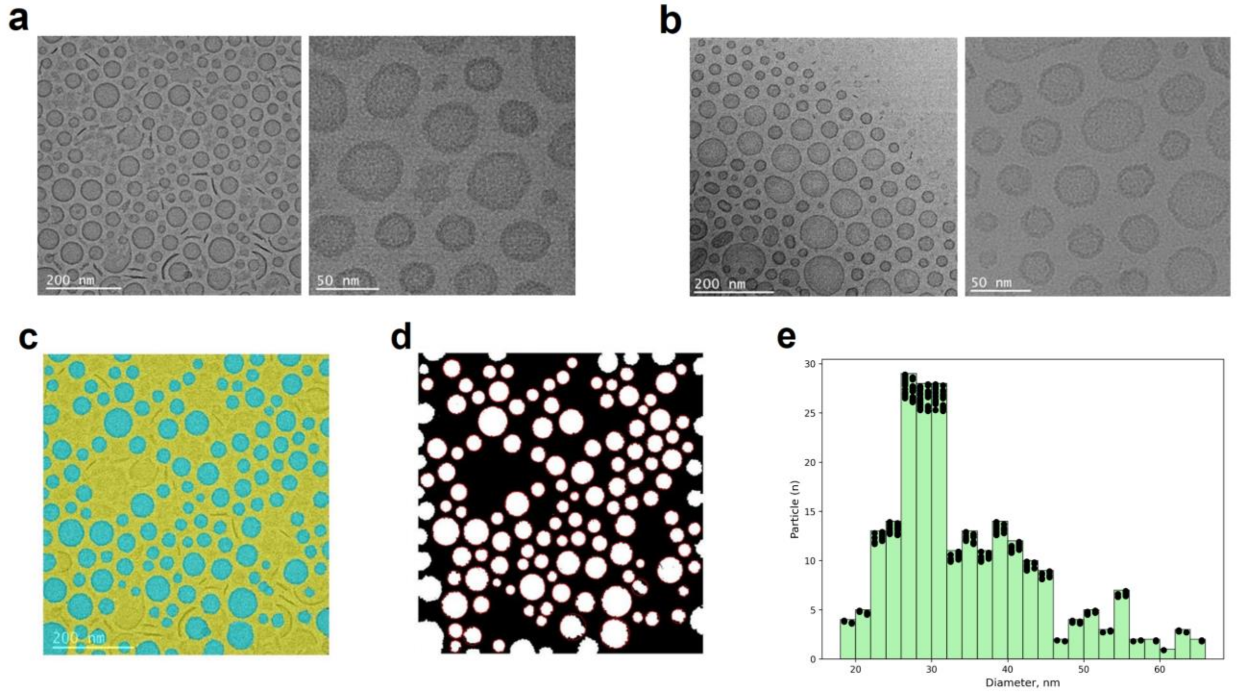

Supplementary Figure 2. Cryo-transmission electron microscopy (cryo-TEM) analyses for the LNP formulations. FTR-LNPs and empty LNPs were synthesized by the thin-film method**. a** FTR-LNPs and **b** empty LNPs under cryo-TEM. Scale bars = 200 nm (left) and 50 nm (right). **c, d** Image segmentation using AI and contour detection for FTR-LNP size distribution measurement. Scale bar = 200 nm. **e** Size distribution histogram corresponding to the cryo-TEM measurements using image processing.

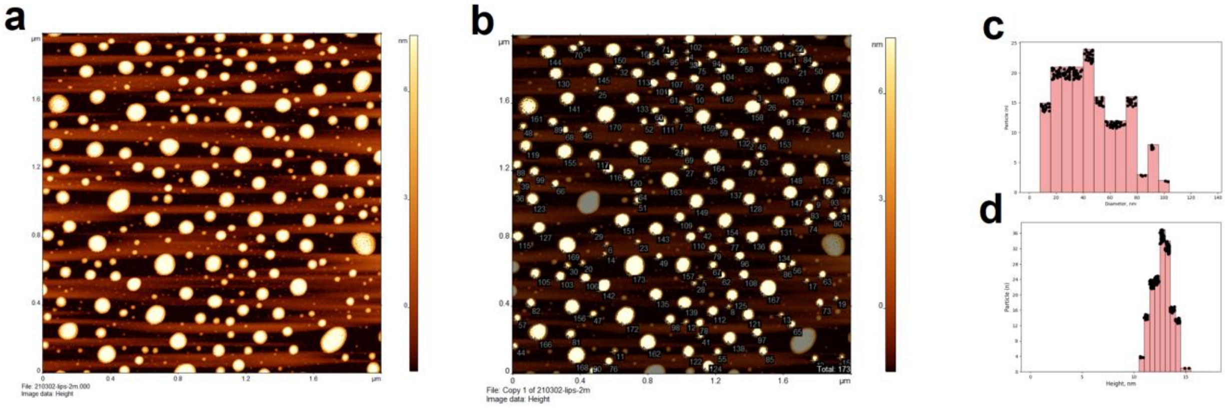

Supplementary Figure 3. AFM of FTR-LNPs synthesized by thin film hydration. a FTR-LNP distribution on mica substrate. **b** Individual FTR-LNP size measurements with software. **c, d** Corresponding histograms of FTR-LNP size distribution in diameter (nm) and particle heights to represent the particles' morphological features.

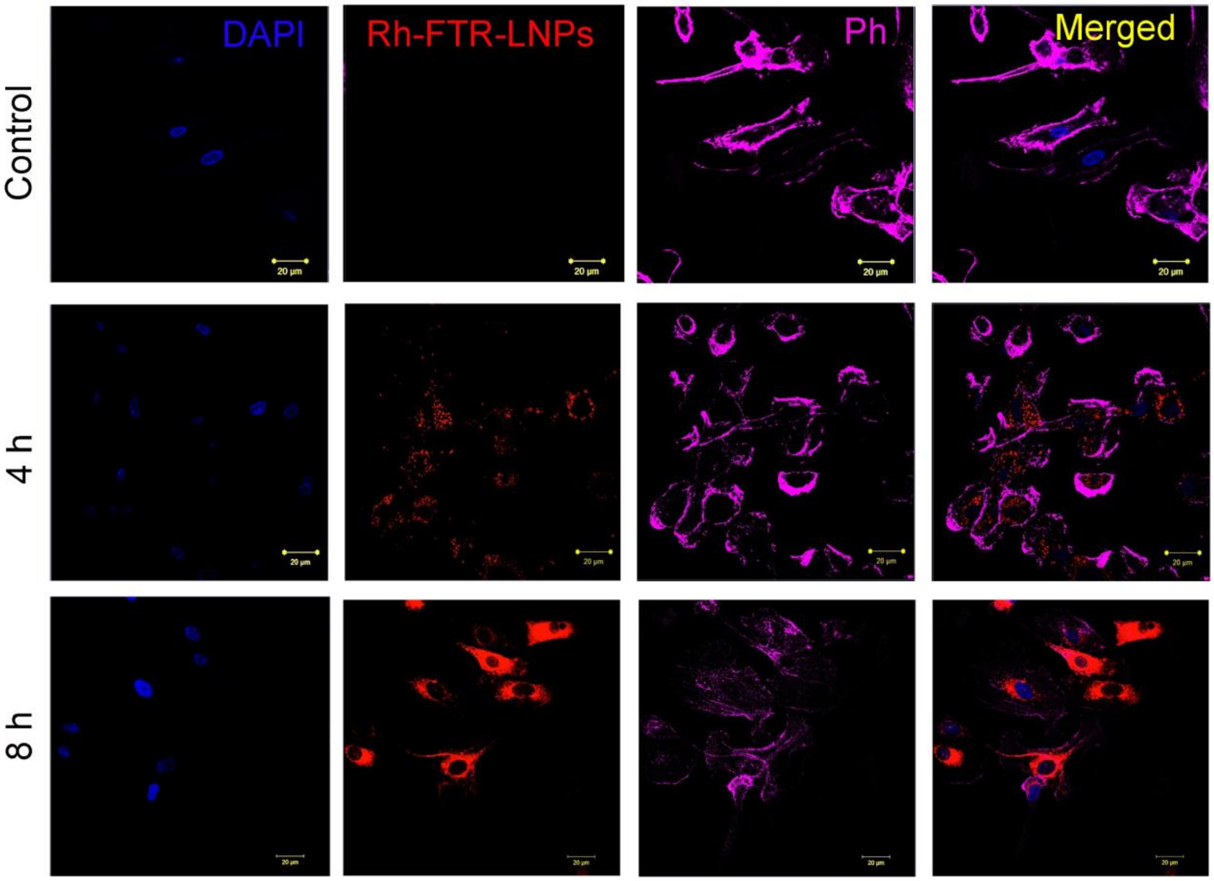

Supplementary Figure 4. Rh-FTR-LNP cell trafficking assayed by confocal microscopy. Representative images of MDM treated with 100 μM Rh-FTR-LNP (red; synthesized by thin-film hydration) after incubation for 4 and 8 h at 37 °C. Nuclei were stained with DAPI (blue). The cell membrane was visualized by staining for F-actin using Alexa Fluor™ 680 Phalloidin (magenta). FTR-LNPs (red) were found in the cytoplasm of MDM. Untreated cells served as negative control. Scale bars = 20 μm. *N* = 3 biological replicates.
