# Supplementary material for: Development of an extended action fostemsavir lipid nanoparticle
Source: Commun Biol. 2024 Jul 30;7:917. doi: 10.1038/s42003-024-06589-5 (PMC11289258; doi:10.1038/s42003-024-06589-5)
Supplement: Supplementary file 2 — Description of Additional Supplementary Materials [file 42003_2024_6589_MOESM2_ESM.pdf]

Description of Additional Supplementary Files

File name: Supplementary Data

Description: The source data of the graphs
