# Supplementary material for: Development of an extended action fostemsavir lipid nanoparticle
Source: Commun Biol. 2024 Jul 30;7:917. doi: 10.1038/s42003-024-06589-5 (PMC11289258; doi:10.1038/s42003-024-06589-5)
Supplement: Supplementary file 4 — Reporting Summary [file 42003_2024_6589_MOESM4_ESM.pdf]

nature portfolio

Corresponding author(s): Howard Gendelman

Last updated by author(s): Jun 14, 2024

Reporting Summary

Nature Portfolio wishes to improve the reproducibility of the work that we publish. This form provides structure for consistency and transparency in reporting. For further information on Nature Portfolio policies, see our Editorial Policies and the Editorial Policy Checklist.

Please do not complete any field with "not applicable" or n/a. Refer to the help text for what text to use if an item is not relevant to your study. For final submission: please carefully check your responses for accuracy; you will not be able to make changes later.

Statistics

For all statistical analyses, confirm that the following items are present in the figure legend, table legend, main text, or Methods section.

n/a	Confirmed	
☐	☒	The exact sample size (*n*) for each experimental group/condition, given as a discrete number and unit of measurement
☐	☒	A statement on whether measurements were taken from distinct samples or whether the same sample was measured repeatedly
☒	☐	The statistical test(s) used AND whether they are one- or two-sided<br>*Only common tests should be described solely by name; describe more complex techniques in the Methods section.*
☒	☐	A description of all covariates tested
☒	☐	A description of any assumptions or corrections, such as tests of normality and adjustment for multiple comparisons
☒	☐	A full description of the statistical parameters including central tendency (e.g. means) or other basic estimates (e.g. regression coefficient) AND variation (e.g. standard deviation) or associated estimates of uncertainty (e.g. confidence intervals)
☒	☐	For null hypothesis testing, the test statistic (e.g. *F*, *t*, *r*) with confidence intervals, effect sizes, degrees of freedom and *P* value noted<br>*Give P values as exact values whenever suitable.*
☒	☐	For Bayesian analysis, information on the choice of priors and Markov chain Monte Carlo settings
☒	☐	For hierarchical and complex designs, identification of the appropriate level for tests and full reporting of outcomes
☒	☐	Estimates of effect sizes (e.g. Cohen's *d*, Pearson's *r*), indicating how they were calculated

Our web collection on statistics for biologists contains articles on many of the points above.

Software and code

Policy information about availability of computer code

Data collection	Not applicable
Data analysis	The custom computer code used in the "LNP Size Distribution by Artificial Intelligence (AI)" section of this manuscript is publicly available. The code can be accessed at the following GitHub repository: https://github.com/asadayon/LNP-size-Distribution. The repository is archived with a DOI provided by Zenodo: 10.5281/zenodo.11540200, ensuring permanent availability.

For manuscripts utilizing custom algorithms or software that are central to the research but not yet described in published literature, software must be made available to editors and reviewers. We strongly encourage code deposition in a community repository (e.g. GitHub). See the Nature Portfolio guidelines for submitting code & software for further information.

Data

Policy information about availability of data

All manuscripts must include a data availability statement. This statement should provide the following information, where applicable:

- Accession codes, unique identifiers, or web links for publicly available datasets
- A description of any restrictions on data availability
- For clinical datasets or third party data, please ensure that the statement adheres to our policy

The source data of the graphs are provided as a Supplementary Data file. The data that support the findings of this study are available from the corresponding authors upon reasonable request.

Research involving human participants, their data, or biological material

Policy information about studies with human participants or human data. See also policy information about sex, gender (identity/presentation), and sexual orientation and race, ethnicity and racism.

Reporting on sex and gender	Any report on sex, age, gender, or "other" information linked to the donor monocytes is strickly prohibited by the IRB.
Reporting on race, ethnicity, or other socially relevant groupings	n/a
Population characteristics	n/a
Recruitment	n/a

Ethics oversight “Research involving human participants, their data, or biological material is as follows: “The acquisition of human blood cells through leukapheresis from HIV-1/2 and hepatitis seronegative donors was approved by the UNMC Institutional Review Board. All ethical regulations relevant to human research participants were followed. All human donors provided written informed consent for collecting and using their blood in this study.

Note that full information on the approval of the study protocol must also be provided in the manuscript.

Field-specific reporting

Please select the one below that is the best fit for your research. If you are not sure, read the appropriate sections before making your selection.

☒ Life sciences ☐ Behavioural & social sciences ☐ Ecological, evolutionary & environmental sciences

For a reference copy of the document with all sections, see nature.com/documents/nr-reporting-summary-flat.pdf

Life sciences study design

All studies must disclose on these points even when the disclosure is negative.

Sample size	No sample size calculation was performed.
Data exclusions	No data were excluded from the analyses
Replication	All attempts on replication was successful
Randomization	Allocation was random
Blinding	Not applicable

Reporting for specific materials, systems and methods

We require information from authors about some types of materials, experimental systems and methods used in many studies. Here, indicate whether each material, system or method listed is relevant to your study. If you are not sure if a list item applies to your research, read the appropriate section before selecting a response.

Materials & experimental systems

n/a	Involved in the study	
☐	☒	Antibodies
☐	☒	Eukaryotic cell lines
☒	☐	Palaeontology and archaeology
☐	☒	Animals and other organisms
☒	☐	Clinical data
☒	☐	Dual use research of concern
☒	☐	Plants

Methods

n/a	Involved in the study	
☒	☐	ChIP-seq
☐	☒	Flow cytometry
☒	☐	MRI-based neuroimaging

Antibodies

Antibodies used	The primary antibodies Rab7 (H-50) (sc-10767) and Lamp1 (H4A3) (sc-20011) used in the study were obtained from Santa Cruz Biotechnology, Inc. (Dallas, TX, USA) and Alexa Fluor™ 488 goat anti-rabbit (A11008) secondary antibody was purchased from Thermo Fischer Scientific. Monoclonal mouse anti-human HIV-1p24 (clone Kal-1) and the polymer-based HRP-conjugated anti-mouse Envision secondary was purchased from Dako (Carpinteria, CA, USA)

Validation	Data provided in manuscript

Eukaryotic cell lines

Policy information about cell lines and Sex and Gender in Research

Cell line source(s)	Monocytes were acquired through leukapheresis from donors who tested negative for HIV-1/2 and hepatitis B.
Authentication	*Describe the authentication procedures for each cell line used OR declare that none of the cell lines used were authenticated.*
Mycoplasma contamination	*Confirm that all cell lines tested negative for mycoplasma contamination OR describe the results of the testing for mycoplasma contamination OR declare that the cell lines were not tested for mycoplasma contamination.*
Commonly misidentified lines (See ICLAC register)	Not applicable

Animals and other research organisms

Policy information about studies involving animals; ARRIVE guidelines recommended for reporting animal research, and Sex and Gender in Research

Laboratory animals	Male Balb/c mice (12 weeks old, 25 g body weight) were obtained from Charles River Laboratories (Stilwell, KS, USA)
Wild animals	Not applicable
Reporting on sex	Not applicable
Field-collected samples	Not applicable
Ethics oversight	Animals were accommodated in the UNMC Comparative Medicine animal facility adhering to the guidelines outlined in the Guide for the Care and Use of Laboratory Animals (National Research Council of the National Academies, 2011). This ensured the ethical treatment and utilization of laboratory animals in experimental research, as per protocol 22-029-08-EP. The housing followed the guidelines outlined by the Association for Assessment and Accreditation of Laboratory Animal Care (AAALAC). Approval for animal experimental protocols was obtained from the UNMC Institutional Animal Care and Use Committee (IACUC), ensuring alignment with the National Institutes of Health's standards and ethical guidelines for treating laboratory animals in research.

Note that full information on the approval of the study protocol must also be provided in the manuscript.

Plants

Seed stocks	Not applicable
Novel plant genotypes	Not applicable
Authentication	Not applicable

Flow Cytometry

Plots

Confirm that:

☒ The axis labels state the marker and fluorochrome used (e.g. CD4-FITC).

☒ The axis scales are clearly visible. Include numbers along axes only for bottom left plot of group (a 'group' is an analysis of identical markers).

☒ All plots are contour plots with outliers or pseudocolor plots.

☒ A numerical value for number of cells or percentage (with statistics) is provided.

Methodology

Sample preparation	Based on the MTT result, 1.0 x 106 MDM per well were treated with 200 µM Rh-LNP1 in 12-well plates for 4 h and 10 h. After treatment, cells were washed with PBS three times. 500 µL PBS was added and cells were collected in flow tubes. Cells were

centrifuged at 400 x g at room temperature. The supernatant was discarded, and cells stained with LIVE/DEAD™ Fixable Blue Dead Cell Stain Kit (ThermoFischer Scientific) and fixed with FACs fix (2% v/v formalin (Sigma-Aldrich) in PBS). The cells were analyzed by flow cytometry with appropriate controls. The fluorochrome combinations used for this experiment were Rhodamine B and LIVE/DEAD™ Fixable Blue stain. Compensation was performed using single-stained cells and unstained cells.

Instrument	BD LSRFortessa™ SORP Flow Cytometer (BD Biosciences, San Jose, CA, USA).
Software	BD FACSDiva 8.0 software (BD Biosciences, San Jose, CA, USA).
Cell population abundance	Not applicable
Gating strategy	The gating tree was set as follows: FSC/SSC (represents the distribution of cells in the light scatter based on size and complexity, respectively) to FSC-A/FSC-H (excludes events which could be more than single cells) to LIVE gate (Fixable Blue negative, which represents viable cells) to FSC-A/Rhodamine B or Rhodamine B as a histogram. Biexponential scaling was used. Data was reported as % positive relative to unstained control and single stained LIVE/DEAD™ Fixable Blue stain.

☐ Tick this box to confirm that a figure exemplifying the gating strategy is provided in the Supplementary Information.

This checklist template is licensed under a Creative Commons Attribution 4.0 International License, which permits use, sharing, adaptation, distribution and reproduction in any medium or format, as long as you give appropriate credit to the original author(s) and the source, provide a link to the Creative Commons license, and indicate if changes were made. The images or other third party material in this article are included in the article's Creative Commons license, unless indicated otherwise in a credit line to the material. If material is not included in the article's Creative Commons license and your intended use is not permitted by statutory regulation or exceeds the permitted use, you will need to obtain permission directly from the copyright holder. To view a copy of this license, visit http://creativecommons.org/licenses/by/4.0/
